# Advancing the Resolution of the Sulfur Compound Disproportionation Puzzle in *Dissulfuribacter Thermophilus* S69ᵀ Through Quantitative Label‐Free Comparative Proteomics

**DOI:** 10.1111/1758-2229.70406

**Published:** 2026-08-19

**Authors:** Marilina Fernandez, Barbara Schoepp‐Cothenet, Marie Hemon, Carine Rodrigues‐Machado, Lukas V. F. Novák, Xavier Philippon, Sébastien Laurent, Céline Henry, Karine Alain

**Affiliations:** ^1^ Université Paris‐Saclay, INRAE, AgroParisTech, Micalis Institute Jouy‐en‐Josas France; ^2^ Aix‐Marseille Université, CNRS, BIP‐UMR 7281, IMM, IM2B Marseille France; ^3^ Univ Brest, CNRS, Ifremer, EMR 6002 BIOMEX, IRP 1211 MicrobSea, Unité Biologie et Ecologie Des Ecosystèmes Marins Profonds BEEP Plouzané France

**Keywords:** comparative proteomics, deep‐sea hydrothermal vent, sulfur disproportionation, thermophiles

## Abstract

In deep‐sea hydrothermal vents, sulfur compounds are central for microbial bioenergetics. Despite its significant impact on the global sulfur cycle, anaerobic sulfur compound disproportionation remains the least understood metabolism, with only three bacterial strains having been investigated at the proteomic level for their sulfur disproportionation metabolism to date. Here, we present a comparative proteomics study of the chemolithoautotrophic sulfur‐cycling species *Dissulfuribacter thermophilus* S69ᵀ. Upon thiosulfate disproportionation conditions, proteomics revealed a high abundance of a cytoplasmic thiosulfate reductase AB homologue (PhsAB‐like) that might reductively cleave thiosulfate into sulfide and sulfite. Subsequent steps might involve enzymes from the dissimilatory sulfate reduction, with Apr and Sat potentially functioning in reverse to produce sulfate. Strain S69^T^ exhibited enhanced growth under sulfite/dihydrogen, likely due to both sulfite respiration and disproportionation. Here, a range of new enzymes were more abundant. These included an enzyme containing NrfD, the MolyAB as well as two membranous tetrathionate reductase homologues. Other membranous oxidoreductases such as QmoABC, DsrMKJOP and Complex I, may contribute to the pathway, while autotrophic growth is likely sustained by the Wood–Ljungdahl pathway. These findings highlight the distinct enzymatic features of strain S69ᵀ compared to previously proteomically characterised sulfur‐disproportionating strains, revealing novel enzymes that warrant further analysis.

## Graphical abstract

To date, only three microorganisms have been functionally characterised for sulfur disproportionation. Our work identifies a fourth example and provides novel proteomic insights into its underlying pathways. These findings advance our understanding of microbial sulfur catabolism and highlight the ecological role of *D. thermophilus* S69ᵀ in hydrothermal vent sulfur cycling.

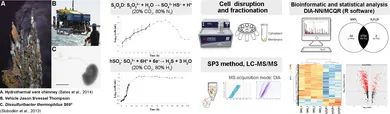

## 1 Introduction

The marine sulfur cycle plays a central role in maintaining the ocean's redox balance and is driven by the multiple oxidation states that sulfur can adopt. Hydrothermal vent ecosystems, which are widespread on the seafloor, are particularly important for the biogeochemical sulfur cycle (Dick 2019). These dynamic and extreme habitats are formed by the mixing of hydrothermal fluids rich in reduced species, particularly sulfide, with oxygenated seawater, creating steep physicochemical gradients that give rise to diverse ecological niches (Kelley et al. 2002; Alain et al. 2022; Wang et al. 2023). These niches host metabolically diverse microbial communities that rely on chemosynthesis, using energy derived from a variety of redox reactions involving inorganic species such as hydrogen, sulfur species (including thiosulfate and sulfite), methane, and iron (Dick 2019; Zeng et al. 2021). In this context, the wide range of sulfur oxidation states (from −2 to +6) enables not only classical unidirectional redox transformations, such as sulfide oxidation or sulfate/sulfite reduction, but also microbial sulfur disproportionation processes (so‐called sulfur dismutation or inorganic fermentation) of sulfur intermediates such as sulfite, thiosulfate, and elemental sulfur (S^0^). In these reactions, a single compound acts as both electron donor and acceptor, and the reaction products are generally sulfate and sulfide (Finster 2008; Wasmund et al. 2017; Slobodkin and Slobodkina 2019), with few exceptions (e.g., *Exiguobacterium* sp. (*Bacillota*) that produces thiosulfate and sulfide from elemental sulfur disproportionation; Wu et al. 2025).

The biochemical reactions associated with thiosulfate and sulfite disproportionation, as well as sulfite reduction, which were addressed in this study, are summarised in Equations ((1), (2), (3)), respectively (Thauer et al. 1977).

(1) $$\begin{array}{c} \text{Thiosulfate disproportionation}:S_{2}{O_{3}}^{2-}+H_{2}O\to {{\mathrm{SO}}_{4}}^{2-}+{\mathrm{HS}}^{-}\\ \;+H^{+}\Delta G^{0}=-21.9\;\mathrm{kJ}\;{\mathrm{mol}}^{-1}S_{2}{O_{3}}^{2-} \end{array}$$

(2) $$\begin{array}{c} \text{Sulfite disproportionation}:4\;{{\mathrm{SO}}_{3}}^{2-}+H^{+}\to 3\;{{\mathrm{SO}}_{4}}^{2-}+{\mathrm{HS}}^{-}\Delta G^{0}\\ \;=-58.9\;\mathrm{kJ}\;{\mathrm{mol}}^{-1}{{\mathrm{SO}}_{3}}^{2-} \end{array}$$

(3) $$\begin{array}{c} \text{Sulfite reduction}:{\mathrm{SO}}_{3}^{2-}+6H^{+}+6e^{-}\to H_{2}S+3\;H_{2}O\;\Delta G^{0}\\ \;=–132\;\mathrm{kJ}\;{\mathrm{mol}}^{-1}{\mathrm{SO}}_{3}^{2-} \end{array}$$

Recent studies demonstrate that anaerobic microbial sulfur disproportionation is sufficiently exergonic to occur in most, if not all, ecological niches of marine hydrothermal vent systems (Alain et al. 2022). Consequently, this metabolism may play a key role in the global biogeochemical sulfur cycle (Fossing and Jørgensen 1990; Jørgensen 1990) and by extension, potentially exert an influence on ocean chemistry and global climate regulation (D'Hondt et al. 2019; Zhou et al. 2025). It might also play a key role in oxygen‐depleted ecosystems with sharp redox fronts (Aronson et al. 2023). Additionally, in certain freshwater and marine sediments, this metabolism has been shown to metabolise over 50% of all thiosulfate, thereby contributing significantly to the sulfur cycle (Jørgensen 1990). Geochemical evidence from sulfur isotope signatures suggests that sulfur disproportionation metabolism is ancient (1300 My old) (Johnston et al. 2005) to very ancient (3490 My old) (Philippot et al. 2007). It may even have existed in primitive Archean ecosystems, possibly preceding sulfate reduction metabolisms, making disproportionation one of the oldest microbial processes on Earth (Finster 2008; Wacey et al. 2011; Wasmund et al. 2017; Novak et al. 2026).

Despite its evolutionary and ecological importance, microbial sulfur disproportionation remains a fairly poorly understood metabolism, particularly in hydrothermal ecosystems, where several dozens of sulfur‐disproportionating bacterial strains have been isolated to date (e.g., (Slobodkin et al. 2013, 2016; Slobodkina et al. 2017; Frolova et al. 2018; Slobodkin and Slobodkina 2019; Hashimoto et al. 2022; Yvenou et al. 2022; Hemon et al. 2025)). Multiple genes and gene clusters, notably the *moly* cluster (composed of molybdopterin oxidoreductase subunits A and B, and a TorD/DmsD chaperone) and the *YTD* cluster (consisting of a *yedE*‐related gene, a *tusA* sulfurtransferase, a *dsrE*‐related gene and 2–3 conserved proteins), have been proposed to be involved in microbial sulfur disproportionation (Allioux et al. 2022; Novak et al. 2026). However, recent in vitro studies demonstrated that the DsrE‐like protein, along with two hypothetical proteins within the YTD cluster, forms a DsrEFH Type II complex with sulfurtransferase activity, which is not restricted to sulfur‐disproportionators but may play a role in the oxidative branch of sulfur disproportionation in specific sulfur‐disproportionating organisms (Plum‐Jensen et al. 2026).

The enzymatic pathways underpinning anaerobic sulfur disproportionation are not yet fully resolved, and comparative genomics clearly showed that this metabolism is performed by multiple distinct pathways in *Bacteria* (Finster 2008; Mardanov et al. 2016; Slobodkin and Slobodkina 2019; Allioux et al. 2020; Novak et al. 2026). Functional insights remain scarce, with only three studies investigating sulfur disproportionation at the proteomic level reported to date, two involving representatives of the phylum *Desulfobacterota* (Hashimoto et al. 2022; Sorokin et al. 2025) and one involving representatives of the phylum *Campylobacterota* (Florentino et al. 2019). Studies conducted on *Desulfobacterota* strains (i.e., *Desulfurivibrio dismutans* AMeS2 and *Desulfolithobacter dissulfuricans* GF1^T^, class *Desulfobulbia*) have suggested that the oxidative branch of this metabolism may proceed via a reversal of the canonical sulfate reduction pathway and that the reductive branch may involve a periplasmic facing polysulfide reductase (PsrABC) (RS06465‐06490) or an enzyme homologous to a tetrathionate reductase (TtrBCA) (GF1_03930; GF1_03940; GF1_03950) (Hashimoto et al. 2022; Sorokin et al. 2025). Under thiosulfate disproportionation conditions, proteins of the *YTD* gene cluster showed increased abundance in *Desulfolithobacter dissulfuricans* GF1^T^ (Hashimoto et al. 2022). A study conducted on *Desulfurella amilsii* TR1^T^ (*Campylobacterota*), not encoding the sulfate reduction pathway, suggested a role in disproportionation of S^0^ of a rhodanese‐like sulfurtransferase (DESAMIL20_2007) and of a sulfide:quinone reductase (DESAMIL20_2009) (Florentino et al. 2019).

The present study focused on a strain belonging to a genus and class (*Dissulfuribacteria*) of *Desulfobacterota* other than those on which proteomic studies of sulfur disproportionation have been conducted before. *Dissulfuribacter thermophilus* strain S69^T^, which was the subject of a comparative proteomic analysis, is notable for its ability to grow by disproportionation of sulfite, thiosulfate, and elemental sulfur (Slobodkin et al. 2013). Here, we investigated the proteomic landscape of this strain during growth via thiosulfate disproportionation (hereafter designated S_2_O_3_D) and in a hydrogenotrophic sulfite‐containing condition (hereafter designated hSO_3_), and examined the associated sulfur chemical intermediates. Using a combination of cultivation, proteomic, and analytical approaches, we provide novel insights into the physiological properties and enzymatic repertoire underlying these sulfur metabolism pathways.

## 2 Materials and Methods

### 2.1 Bacterial Strain and Growth Cultivation

*Dissulfuribacter thermophilus* S69^T^(=DSM 25762^T^), a thermophilic, anaerobic, chemolithoautotrophic bacterium, was isolated from a hydrothermal vent chimney from the Valu Fa Ridge, in the Lau back‐arc basin, South‐West Pacific (Slobodkin et al. 2013).

S_2_O_3_D was investigated in 5 L bottles containing 500 mL of mineral medium containing a synthetic buffer and adjusted to pH 6.8, prepared without the sulfide scavenger Fe(OH)_3_. The mineral medium composition (in g/L) was as follows: MgCl_2_·6H_2_O, 4.4; NaCl, 25; NH_4_Cl, 0.33; KCl, 0.5; CaCl_2_·2H_2_O, 0.5; KH_2_PO_4_, 0.33; and PIPES (1,4‐Piperazinediethanesulfonic acid) buffer, 6.92, supplemented with vitamins and trace elements as described elsewhere (Slobodkin et al. 2012) and with 30 mM Na_2_S_2_O_3_. The medium was sterilised by autoclaving under standard conditions (121°C, 15 psi, 20 min). Cultures were incubated statically under an N_2_/CO_2_ (80:20) atmosphere at 60°C for 42 h. Incubations under hydrogenotrophic sulfite condition (hSO_3_) were conducted in the same mineral medium. After sterilisation, sterile Na_2_SO_3_ was aseptically added from a stock solution to a final concentration of 5 mM. Cultures were incubated statically at 60°C for 12 h under an H_2_/CO_2_ (80:20, v/v) atmosphere. All experiments were performed in four biological replicates.

For each condition, cells were cultivated in the targeted medium for three consecutive transfers. Subsequently, four independent pre‐cultures were established for each condition and incubated for 24 h under hydrogenotrophic sulfite conditions and for 72 h under thiosulfate disproportionation conditions. At the end of the incubation period, pre‐cultures reached cell densities of 8.66 × 10^7^ cells/mL and 3.78 × 10^7^ cells/mL, respectively, and were used as inocula for the experimental cultures. Iron(III) hydroxide (Fe(OH)_3_) (32 mM) was added exclusively to the pre‐cultures for thiosulfate disproportionation. Experimental cultures were inoculated at 1:20 (thiosulfate) and 1:30 (sulfite) dilution ratios, after reducing the medium with Na_2_S (0.33 mM). Growth curves were characterised in independent experiments over 104 h for sulfite reduction and 120 h for thiosulfate disproportionation (Supplementary Figure S1).

As the PIPES buffer contains sulfur, control cultures without added substrate (SO_3_^2−^ or S_2_O_3_^2−^) were carried out to determine whether any growth could be attributed to PIPES. Uninoculated abiotic controls were also performed for each condition.

### 2.2 Microbiological and Chemical Analysis

At predetermined time points along the growth curve—2 h for hydrogenotrophic sulfite condition and 4 h for thiosulfate disproportionation—aliquots were collected for bacterial cell counting, pH monitoring, and quantification of substrate and metabolic product concentrations.

Bacterial growth was quantified by direct cell counting under an epifluorescence microscope following fixation with glutaraldehyde, staining with acridine orange, and filtration onto 0.2 μm polycarbonate membranes. Cells were counted under UV light (×100 objective) in at least 20 randomly selected grid squares (0.0025 μm^2^ each), with a minimum of 300 cells counted per sample. More details are provided in the Supporting Information.

For anion analysis, especially sulfate, sulfite, and thiosulfate, 1 mL of culture was centrifuged at 15,000 g for 2 min, and the supernatant was filtered through a Millex‐GV filter (0.22 μm, PVDF, 13 mm, Millipore, Millipore Corporation, MA, USA) and diluted one‐tenth with filtered MilliQ water into pyrolyzed Chromacol vials (Thermo Scientific, Loughborough, UK). Aliquots were stored at −20°C until analysis by ion chromatography (IC), as detailed in Supporting Information.

Gaseous hydrogen sulfide (H_2_S) production was initially intended to be analysed by gas chromatography. However, due to equipment unavailability, the protocol was adapted at the last minute to determine dissolved hydrogen sulfide (H_2_S) concentrations spectrophotometrically using the protocol elsewhere (Cline 1969). For this purpose, 2 mL of culture was centrifuged at 15,000 g for 2 min. The supernatant was immediately transferred to vials containing 125 μL of 10% (w/v) zinc acetate (Sigma‐Aldrich) to precipitate sulfide as ZnS. Although an H_2_S signal was detected in these cultures, precise quantification was not possible due to interference from sulfite and thiosulfate present in the medium. Consequently, the concentration of the missing sulfur compounds, primarily H_2_S/HS^−^ based on previous experiments (Slobodkin et al. 2013), was deduced from the normalised concentrations of sulfur species measured by IC to ensure a balanced sulfur species budget. However, the presence of elemental sulfur, polysulfides, tetrathionate, or other sulfur intermediates that were not monitored cannot be excluded.

### 2.3 Genome Analysis

The genome sequence of the studied organism was made publicly available (NCBI GenBank accession GCA_978936825.1). The genome was analysed on the MicroScope Microbial Genome Annotation and Analysis Platform (MaGe) v3.17.3 (Vallenet et al. 2020) using KEGG and BioCyc databases, along with the following software tools: SignalP 6.0 (Teufel et al. 2022), InterPro v108.0 (Blum et al. 2025), Pfam (Paysan‐Lafosse et al. 2025), DiSCo (Neukirchen and Sousa 2021), and DeepTMHMM (Hallgren et al. 2022). Predicted coding sequences (CDSs) were functionally annotated by comparison against the UniProtKB reference proteomes and the Swiss‐Prot database (The UniProt Consortium 2025). Additionally, where necessary, further genes were manually identified and annotated using (implemented in the MaGe platform) and the hmmsearch tool of HMMER 3.4 (Eddy 2011).

### 2.4 Proteomic Analysis

#### 2.4.1 Biomass Collection, Cell Fractionation and Protein Extraction

Cells grown under S_2_O_3_D and hSO_3_ conditions were harvested at mid‐exponential phase (42 h and 12 h, respectively), washed, and stored at −80°C (see Supporting Information). Pellets were resuspended in lysis buffer (50 mM Tris–HCl, pH 8.0) supplemented with phosphatase and protease inhibitors. Cell lysis was performed using a high‐pressure disruptor, and cytoplasmic and membrane protein fractions were obtained by centrifugation (257,000 × g for 50 min at 4°C). The membrane fraction was solubilised in DUTT buffer, sonicated, and clarified by centrifugation (7440 × g for 2 min at 4°C). Protein concentrations were determined using the Bradford assay with BSA as a standard (Bradford 1976). For additional details, see Supporting Information.

#### 2.4.2 Mass Spectrometry Analysis

Sample preparation for nano‐flow liquid chromatography coupled to tandem mass spectrometry (nanoLC‐MS/MS) followed the SP3 protocol (Hughes et al. 2019; Hansen et al. 2025), as detailed in the Supporting Information. LC–MS/MS analysis was conducted at the PAPPSO platform (http://pappso.inra.fr) using a timsTOF Pro (trapped ion mobility spectrometry–time‐of‐flight) mass spectrometer coupled to a nanoElute LC system (Bruker, Daltonics, Gmbh). Tryptic peptides were first loaded onto an Acclaim PepMap 100 C18 trap column (100 μm i.d. × 20 mm, 5 μm particle size, 100 Å pore size; Thermo Scientific), and subsequently separated on an Aurora C18 analytical column (25 cm × 75 μm i.d., 1.6 μm particle size; IonOpticks, Victoria, Australia). Peptides were eluted through the nanoLC system using the following gradient at a flow rate of 250 nL/min: 2% buffer B (100% acetonitrile –ACN‐ and 0.1% formic acid) within 0–42 min, 13% buffer B in 42–65 min, and 20% buffer B in 65–70 min, 30% buffer B in 70–75 min and 85% over the last 12 min. Peptides were acquired using an optimised data‐independent acquisition (dia‐PASEF) mode.

For that, the electrospray voltage was set to 1600 V, with a gas flow rate of 3.0 L/min at 180°C. Full MS data were acquired in a mass range of 100–1700 *m*/*z* and an ion mobility range of 0.70–1.20 (1/K0). DIA was performed using 29 windows covering the 100–1700 *m*/*z* range, with ramp times of 100 ms, resulting in a total cycle time of 1.27 s. Collision energy was ramped according to ion mobility, from 20 eV at 0.6/K0 to 59 eV at 1.6 1/K0 (V·s/cm^2^).

DIA‐MS raw files were analysed using DIA‐NN (version 1.8.1) (Demichev et al. 2020) against an *in silico* predicted spectral library based on the *D. thermophilus* S69^T^ UniProt database (2462 entries, release 24‐04‐2024) and a contaminant database (Tables [Link], [Link]). The protease was set to ‘Trypsin/P’ with two missed cleavages allowed. Other data analysis parameters included a precursor *m*/*z* range of 300–1800, fragment *m*/*z* range of 200–1800, precursor charge states of 2–4, and peptide lengths between 7 and 30 amino acids. Fixed modifications (maximum of one per peptide) included N‐terminal methionine excision and carbamidomethylation of cysteine, and oxidation of methionine was set as a variable modification. Mass accuracy was set to 15 ppm for both precursor and fragment ions. Protein inference was performed with a 1% false discovery rate (FDR) at both the protein and peptide levels. Retention time‐dependent cross‐run normalisation was applied using DIA‐NN's built‐in algorithm. The identified proteins were identified with a minimum of one peptide per protein.

#### 2.4.3 Data Processing and Statistical Analysis

Relative quantification of protein abundances was achieved using extracted ion chromatograms (XICs), calculated as the summed MS2 fragment ion intensities from all peptides assigned to each protein (Valot et al. 2011). To estimate total cellular protein abundance, LFQ intensities from the soluble and membrane fractions were mathematically summed for each protein. This approach was adopted because these fractions correspond to operationally defined cellular fractions rather than strictly quantitative partitions. Relying on a single fraction could lead to an underestimation of protein abundance. However, variations in extraction efficiency, peptide recovery, and ionisation behaviour across fractions may introduce quantitative biases; therefore, summed LFQ values should be interpreted as estimates of total protein abundance rather than absolute measurements. Data post‐processing and statistical analysis were conducted with the R package MCQR v1.0.2 (Balliau et al. 2025) (Supplementary Table S4).

After chromatographic quality assessment and reproducibility checks among biological replicates, four hSO_3_ and three S_2_O_3_D replicates were retained for further analysis (Figures [Link], [Link], Supporting Information S1). Data filtering included: (i) removal of peptides with retention time variation > 30 s; (ii) exclusion of peptides with *m*/*z* values detected within retention time windows of 250–1125 s and 4625–5000 s; (iii) normalisation of peptide intensities based on retention time median (Lyutvinskiy et al. 2013) (Supplementary Figure S2); (iv) removal of shared peptides; (v) exclusion of peptides with > 10% missing values across all MS runs; (vi) imputation of missing peptide intensities by replacing values with the minimum observed abundance for each protein across the dataset using the K‐nearest neighbours (KNN) method; and (vii) removal of proteins with abundance variation < 1.5 between metabolisms. The hypothesis test was performed and proteins with adjusted *p‐*values (*p*_adj_) ≤ 0.05 were considered significantly variable (Supplementary Table S5). Biological interpretation was supported using bioinformatics tools including PSORT v3.0.2, STRING v.12.0, and IPhat3 v.3 (Yu et al. 2010; Darzi et al. 2018; Szklarczyk et al. 2023). The mass spectrometry proteomic data have been deposited to the ProteomeXchange Consortium via the partner repository PRIDE, under the dataset identifier [PXD069670].

## 3 Results and Discussion

### 3.1 Bacterial Growth Occurred Under Both Culture Conditions

The metabolic properties of strain S69^T^ relevant to this study included its previously demonstrated ability to disproportionate thiosulfate and sulfite to sulfate and sulfide (Slobodkin et al. 2013), as well as its ability to respire sulfite with H2 as an electron donor and CO_2_ as a carbon source, as shown in earlier experiments (unpublished data).

**FIGURE 1:**
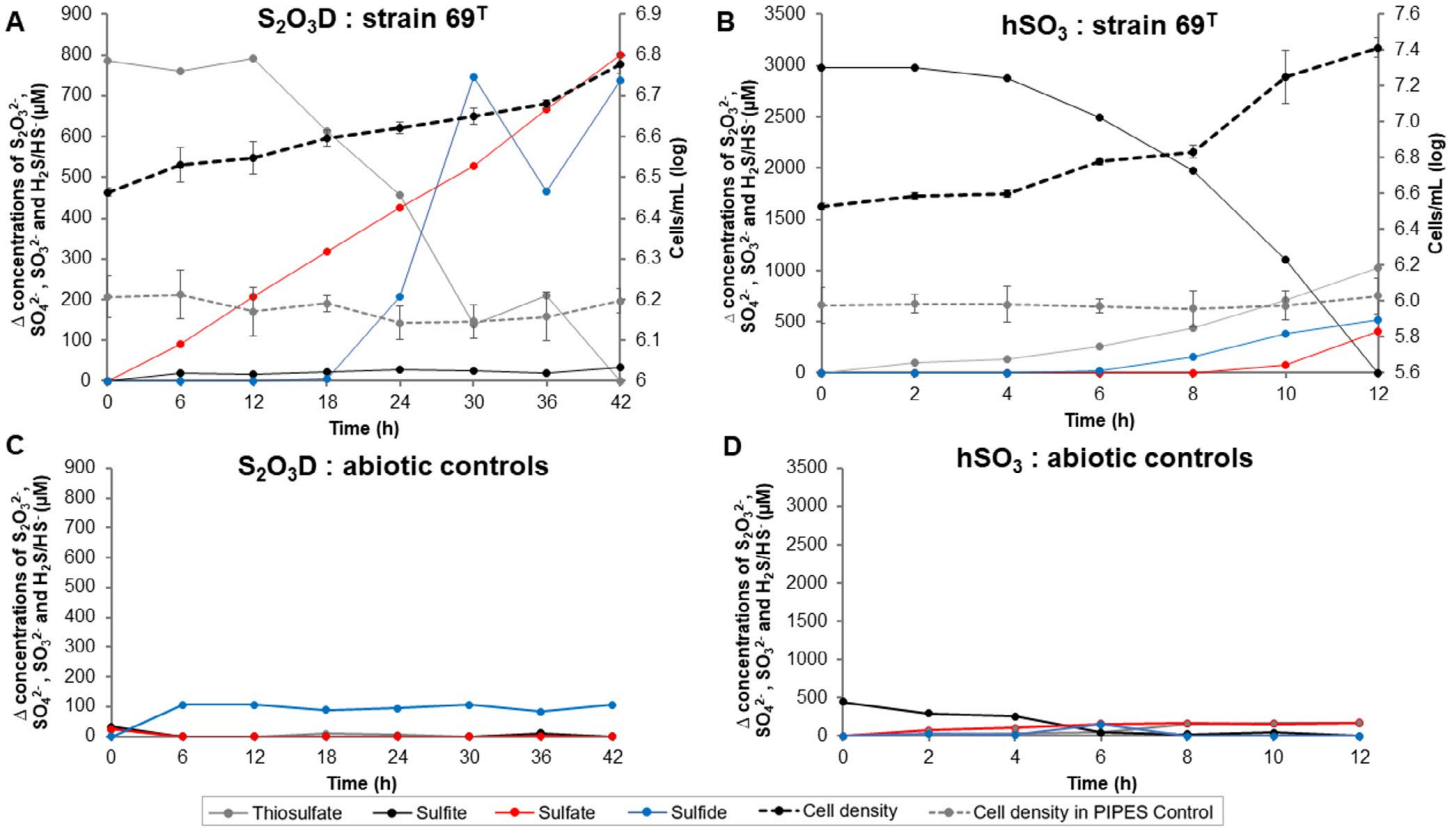
Growth of strain S69^T^ under thiosulfate disproportionation conditions (S_2_O_3_D) (A) and in hydrogenotrophic sulfite‐containing medium (hSO_3_) (B), alongside temporal changes in sulfur species concentrations for each condition. The sampling time point for proteomic analyses was at 42 h and 12 h for each metabolism, respectively. In addition, abiotic controls and inoculated PIPES controls without substrates were conducted in parallel. Cell density in PIPES controls prepared without added substrates is also presented in diagrams A and B. Temporal changes in sulfur species concentrations under S_2_O_3_D (C) and hSO_3_ (D) conditions are shown for abiotic controls (PIPES controls are not shown, as no formation of the measured sulfur species was observed in PIPES controls prepared without substrates).

Strain S69T grew in the presence of sulfite and dihydrogen (hSO3), with cell density increasing from 3.38 × 10^6^ ± 1.37 × 10^5^ to 2.00 × 10^7^ ± 3.16 × 10^6^ cells/mL after 12 h (μ = 0.48 h^−1^, paired *t*‐test: t(3) = −21.12, *p* < 0.001), corresponding to a ~5.9‐fold increase of the initial population, and a biological cell yield of 5.57 × 10^3^ cells·mL^−1^·μM^−1^ of sulfite. In contrast, growth under thiosulfate disproportionation conditions (S_2_O_3_D) was weaker, reaching 5.99 × 10^6^ ± 3.02 × 10^5^ cells/mL from an initial 2.91 × 10^6^ ± 6.66 × 10^4^ cells/mL after 42 h (μ = 0.07 h^−1^, paired *t*‐test: t(2) = −33.71, *p* < 0.001) corresponding to a ~2.1‐fold increase in cell density and a growth yield of 3.9 × 10^3^ cells·mL^−1^·μM^−1^ of thiosulfate (Figure 1A,B). The higher specific growth rate observed under hSO3 conditions is consistent with the more favourable energetics associated with sulfite‐based metabolism relative to thiosulfate disproportionation (Finster 2008; Simon and Kroneck 2013). In addition, S_2_O_3_D incubations were carried out without the addition of sulfide scavengers, such as Fe(OH)_3_, which make the reaction more exergonic but make protein extraction much more difficult. In PIPES buffer controls prepared without added substrates, cell numbers remained stable over time for both metabolic conditions, indicating that no growth occurred and confirming that the sulfur present in the buffer was not utilised as a substrate for growth (Figure 1A,B). Furthermore, the concentrations of the various sulfur species exhibited only minor changes in both purely abiotic controls for each condition, and also in inoculated PIPES controls (Figure 1C,D; data not shown). The growth stage (exponential phase) corresponding to the sampling point for proteomic analyses, along with the full growth curves covering all growth phases, are presented in Supplementary Figure S1.

Chemical analyses confirmed that thiosulfate disproportionation metabolism was at work in S_2_O_3_D incubations. Indeed, the consumption of thiosulfate was accompanied by the production of sulfate and sulfide (Figure 1A; Supplementary Figure S1C). The pH remained stable, with values of 6.50 ± 0.03 for S_2_O_3_D and 6.46 ± 0.04 for the hSO_3_ condition over time, likely due to the buffering capacity of PIPES.

In hSO_3_ incubations, sulfite was progressively consumed, and thiosulfate began to accumulate in the medium. Sulfide, the final product of sulfite respiration, was detected only after 6 h of incubation (Figure 1B). The delayed detection of sulfide could be due to the reliability of sulfide measurement at low sulfide concentrations. Since no thiosulfate production was observed in the abiotic controls and considering that thiosulfate accumulation preceded both substantial sulfide accumulation and significant sulfite depletion, its formation could be attributed either (i) to the activity of an inducible, yet uncharacterized enzyme operating in reverse to oxidise sulfite to thiosulfate or (ii) thiosulfate being an intermediate in sulfite disproportionation.

A production of sulfate was also observed after 8 h of incubation, suggesting that sulfite disproportionation also occurred. Based on these observations, it was possible that the strain initially ‐before 8 h‐ favoured sulfite reduction for growth, which is more energetically favourable, and subsequently likely relied on a combination of sulfite reduction and disproportionation, and perhaps thiosulfate reduction and disproportionation, even though thiosulfate consumption was not visible during the incubation period (possibly because it was lower than its production). Under conditions of hydrogenotrophic respiration of thiosulfate, previous studies conducted with the same strain demonstrated that molecular hydrogen consumption was incomplete, but that thiosulfate was also converted into sulfide and sulfate (Slobodkin et al. 2013). However, in the present experiment, no diauxic phase was observed in the growth curve to support this hypothesis of successive metabolic reactions. Studies conducted with some other sulfur‐disproportionating bacterial strains have shown that in the presence of molecular hydrogen, sulfate formation is completely suppressed in cultures containing elemental sulfur or sulfite, and that reduction processes prevail over disproportionation in thiosulfate‐supplemented cultures (Finster 2008). Alternatively, sulfur disproportionation metabolism may have been activated in strain S69^T^ at the start of incubation, as reported in previous experiments with this strain under thiosulfate respiration conditions (Slobodkin et al. 2013). In this case, strain S69^T^ would have been able to carry out respiration and disproportionation processes simultaneously. The prioritisation of metabolic reactions based on their energy yield, with priority given to reactions that produce the most energy, is deeply rooted in the common belief of microbiologists. However, there is increasing evidence that some microorganisms simultaneously carry out two metabolic processes with very different yields. For example, recent studies demonstrated that some bacterial taxa living in habitats with fluctuating oxygen concentrations can perform simultaneous aerobic and anaerobic respirations (e.g., (Keller et al. 2025; Lustermans et al. 2025; Digel et al. 2026)). Strain S69^T^, which originates from a marine hydrothermal environment where chemical species gradients are highly fluctuating, may constantly activate sulfur disproportionation metabolism, as this pathway enables energy production, and thus at least maintenance, using a single sulfur compound as a starting substrate, whereas respiratory metabolisms require two.

### 3.2 Comparative Proteomics Provides Insights Into Energy Conservation Mechanisms

As part of the present study, a complete genome sequence of the studied organism was made publicly available, annotated, and used as a basis for comparative proteomics. To facilitate interpretation of the proteomic data, a brief overview of the genomic organisation of key genes involved in dissimilatory sulfur metabolism is provided in Supplementary Figure S4. The genome contained a canonical reductive sulfur metabolism gene cluster, including genes encoding sulfate adenylyltransferase (Sat), adenylylsulfate reductase (AprAB), the quinone‐interacting membrane‐bound oxidoreductase complex (QmoABC), and the dissimilatory sulfite reductase complex (DsrABCD). Based on sequence analyses, DsrAB, AprAB, and Sat were found to be of the reductive type according to the HMM‐profile‐based DiSCo tool. Several of these genes were found to be organised in conserved operon‐like arrangements, notably the *aprBA*‐*qmoABC* and the *dsrABCD‐dsrMKJOP* clusters. The genome was also found to encode a YTD gene cluster that included a DsrEFH Type II. In addition, genes encoding various molybdopterin oxidoreductases, including the Moly protein cluster (Allioux et al. 2022; Novak et al. 2026) and a tetrathionate reductase homologue in genomic neighbourhood with a porin homologous to the phosphate‐selective porin OprOP were present. The various molybdopterin oxidoreductases encoded in the genome are discussed in the following paragraphs.

**FIGURE 2:**
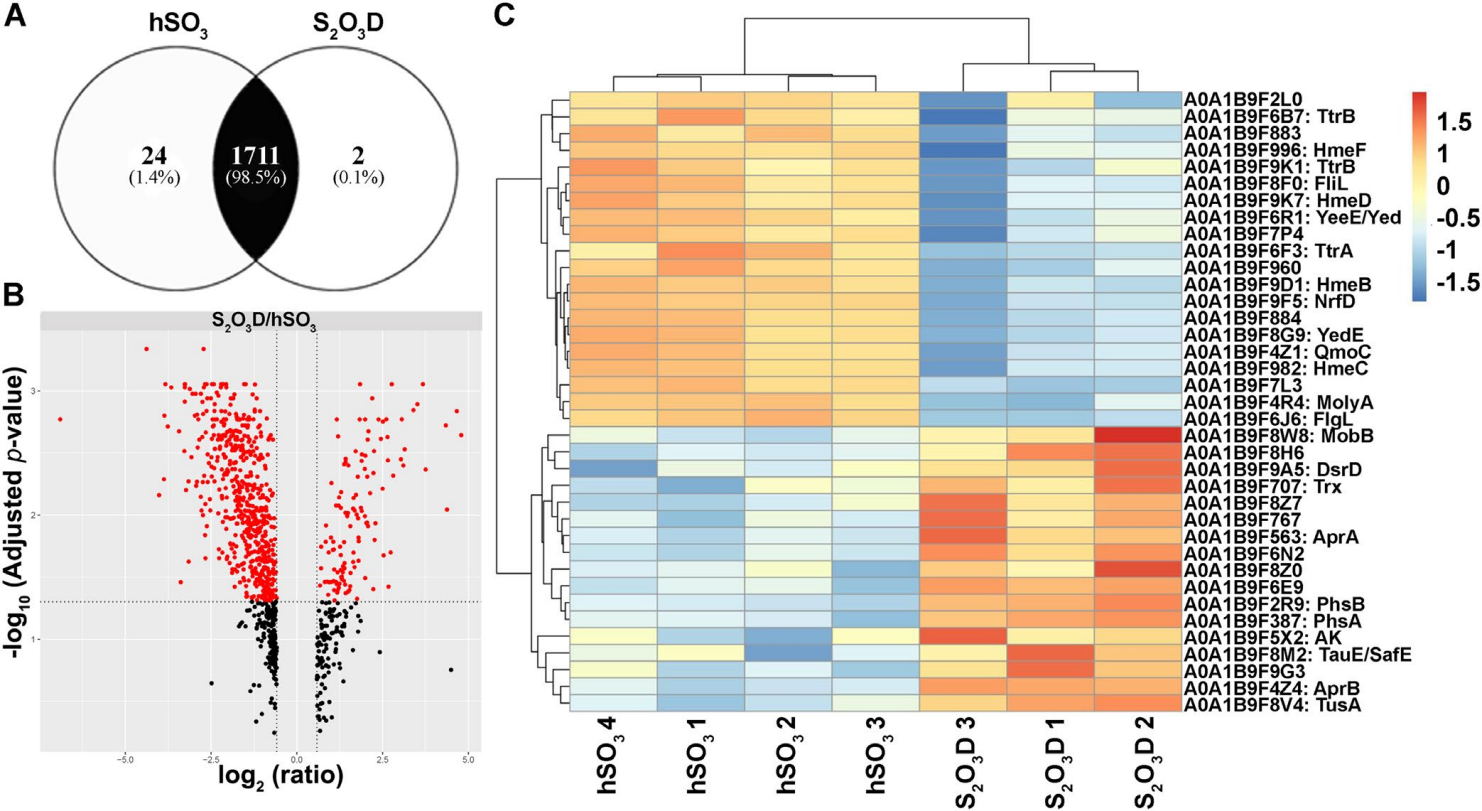
Comparative proteomic analysis of strain S69ᵀ under thiosulfate disproportionation (S_2_O_3_D) and in cultures grown in hydrogenotrophic sulfite‐containing medium (hSO_3_). (A) Venn diagram showing the total number of proteins identified under each condition. (B) Volcano plot highlighting proteins with statistically significant differences in abundance between conditions (S_2_O_3_D vs. hSO_3_). Proteins with a log_2_(fold change) ≥ 0.58 (fold change ≥ 1.5) were considered to show increased abundance, whereas those with a log_2_(fold change) ≤ −0.58 were considered to show decreased abundance. Statistical significance was defined as an adjusted *p*‐value ≤ 0.05 (equivalent to –log_10_(p) ≥ 1.3 on the y‐axis). (C) Heatmap showing the relative abundance of proteins involved in sulfur metabolism (fold change ≥ 1.5; adjusted *p*‐value ≤ 0.05). Proteins with a fold change ≥ 1.5 were selected, and the heatmap was generated using row‐wise scaling of log‐transformed abundances to visualize relative abundance patterns across conditions. A detailed list of these proteins is provided in Tables 2 and 3.

This study examined the presence and relative abundance of key proteins involved in the thiosulfate disproportionation pathway through comparative proteomics, using the proteome of the strain grown on a sulfite‐containing medium under hydrogenotrophic conditions as a reference (Supplementary Tables [Link], [Link]). While interpreting this new proteomic dataset generated under sulfur compound disproportionation conditions, it is important to keep in mind both the general limitations of proteomics—which depend on energetic and physiological states, as well as intracellular redox conditions, and do not provide proof of enzyme activity—and the fact that central sulfur metabolism enzymes may be constitutively expressed regardless of their physiological role. Here, a total of 1737 proteins were identified, representing 71% of the predicted proteome of strain S69ᵀ (1737 of 2462 predicted proteins) (Supplementary Figure 2A). This value is at the upper end of the range reported in the literature for proteomic analyses involving the disproportionation of sulfur compounds (1172 of 3242 predicted proteins (36%) in *Desulfolithobacter dissulfuricans*; 698 of 2088 (33%) in *Desulfurella amilsii*; 1864 of 2491 (75%) in *Desulfurivibrio dismutans*). These results indicated that the fractionation protocol and MS approach provided access to a large fraction of the proteomes. However, it was difficult to infer compartment‐specific information, as a significant proportion of the fractions ultimately proved to be identical. Detailed information on protein identification in the soluble and membrane fractions is provided in Supplementary Figure S5. A total of 717 proteins showed significantly different abundances between S_2_O_3_D and hSO_3_ conditions (fold change ≥ 1.5; adjusted *p* ≤ 0.05), as shown in the volcano plot (Figure 2B) and listed in Supplementary Table S6. As shown in the heatmap (Supplementary Figure S6), and listed in Supplementary Table S7, only 123 proteins were significantly more abundant (log_2_FC ≥ 0.58), under S_2_O_3_D conditions, whereas 594 displayed significantly lower abundance—or conversely higher abundance under hSO_3_ conditions (log_2_FC < −0.58). As illustrated in Figure 2C, several distinct proteins involved in sulfur metabolism were significantly more abundant under either of the conditions, as discussed below. Among the identified proteins, two were exclusively detected under S_2_O_3_D, while 24 were specific to hSO_3_ conditions (Figure 2A). Notably, the molybdopterin oxidoreductase subunit Moly A (A0A1B9F4R4/DITHS69_1420), which forms the MolyAB complex with MolyB (A0A1B9F4Y0/DITHS69_1419), a complex belonging to the DMSO reductase family (Rothery et al. 2008; Hille et al. 2014), was uniquely detected under hSO_3_ conditions. The difficulty in detecting MolyB may be attributed to its physicochemical and analytical properties (e.g., hydrophobicity, ionisation efficiency, or low protein abundance). The increased abundance of MolyA under hSO_3_ conditions suggests a potential role for MolyAB in the initial steps of sulfite reduction or disproportionation.

Other functional studies conducted to date with other *Desulfobacterota* strains have also shown that other molybdopterin oxidoreductases display increased abundance or are highly abundant during sulfur compound disproportionation. For instance, in *Desulfolithobacter dissulfuricans*, the TtrABC‐homologue complex shows significantly increased abundance, and PhsAB‐like is abundant during thiosulfate disproportionation (Hashimoto et al. 2022). Similarly, in *Desulfurivibrio dismutans*, PsrABC‐like shows increased abundance during the disproportionation of elemental sulfur (Sorokin et al. 2025). The remaining exclusive proteins are listed in Supplementary Table S8. Several enzymes involved in sulfur metabolism were very abundant in one or both growth conditions (Table 1). These include different subunits of dissimilatory sulfite reductase DsrAB (highly abundant under both conditions) (A0A1B9F9G6/DITHS69_0299; A0A1B9F9G9/DITHS69_0298) responsible for the reduction of sulfite to sulfide in sulfate/sulfite reduction and responsible for the oxidation of sulfane sulfur to sulfite, in combination with other Dsr proteins, in one sulfur oxidation pathway. Its DsrC co‐substrate (A0A1B9F5A9/DITHS69_1036) was also present under both conditions while not so abundant. Although *dsrAB* expression is known to have a basal level, it can be activated by DsrD, an allosteric activator of DsrAB (Dörries et al. 2016; Wasmund et al. 2017; Ferreira et al. 2022; Neukirchen et al. 2023). Here, DsrD (A0A1B9F9A5/DITHS69_0297) was detected to be significantly more abundant under S_2_O_3_D (Table 2). In addition, several proteins likely involved in the oxidative branch of sulfur disproportionation were found to be abundant. These included the subunits of adenylylsulfate reductase (AprAB) (A0A1B9F563/DITHS69_0899; A0A1B9F4Z4/DITHS69_0898) and sulfate adenylyltransferase (Sat) (A0A1B9F720/DITHS69_0828). AprAB subunits were highly abundant under both conditions, while Sat was abundant only under S_2_O_3_D conditions. Sat is known to participate in the activation of sulfate with ATP to produce adenosine 5′‐phosphosulfate (APS) in sulfate reduction, and to be responsible for the final oxidation step from sulfite to sulfate in sulfur oxidation (Parey et al. 2013). It was also detected under hSO_3_ condition but did not belong to the 20 most abundant proteins. Sulfate production was consistently measured under both conditions. This process was likely associated with ATP formation via substrate‐level phosphorylation (Krämer and Cypionka 1989). In addition, subunits of the adenylylsulfate reductase–associated complex QmoA (A0A1B9F585/DITHS69_0900); QmoB (A0A1B9F5A6/DITHS69_0901) and QmoC (A0A1B9F4Z1/DITHS69_0902) were detected among the most abundant proteins under both studied conditions (Table 1). The QmoABC complex is known to mediate electron transfer from AprAB to the membranous diffusible menaquinone pool or from the membranous menaquinone pool to AprAB, respectively, in sulfur oxidation and sulfate reduction (Ramos et al. 2012). Based on these abundant enzymes, strain S69^T^, like the two other *Desulfobacterota* that have undergone functional analyses to date (Hashimoto et al. 2022; Sorokin et al. 2025), might use the enzymes of the sulfate reduction pathway in reverse for its oxidative branch.

**TABLE 1:** List of the 20 most abundant proteins per metabolism.

ID protein	NCBI gene locus	Description	Symbol	Metabolism	Log_2_ FC (S_2_O_3_D/hSO_3_)	Abundance (mean_log_10_q)
**A0A1B9F563**	**DITHS69_0899**	**Adenylylsulfate reductase alpha‐subunit**	**AprA**	**S**_**2**_ **O**_**3**_ **D**, **hSO**_**3**_	**1.42**	**6.08, 5.65**
**A0A1B9F4Z4**	**DITHS69_0898**	**Adenylylsulfate reductase beta‐subunit**	**AprB**	**S**_**2**_ **O**_**3**_ **D**, **hSO**_**3**_	**2.20**	**6.03, 5.36**
A0A1B9F312	DITHS69_0625	Fibronectin Type III domain protein		S_2_O_3_D		5.94
A0A1B9F799	DITHS69_0791	DUF5666 domain‐containing protein		S_2_O_3_D	1.84	5.70
A0A1B9F788	DITHS69_0152	DUF1104 domain‐containing protein		S_2_O_3_D	4.34	5.62
**A0A1B9F9G9**	**DITHS69_0298**	**Sulfite reductase, dissimilatory‐type subunit beta**	**DsrB**	**S**_**2**_ **O**_**3**_ **D**, **hSO**_**3**_		**5.61, 5.55**
**A0A1B9F5J5**	**DITHS69_1625**	**Glutamine synthetase/glutamate**—**ammonia ligase**	**GlnA**	**S**_**2**_ **O**_**3**_ **D**, **hSO**_**3**_	**0.71**	**5.61, 5.39**
**A0A1B9F9G6**	**DITHS69_0299**	**Sulfite reductase, dissimilatory‐type subunit alpha**	**DsrA**	**S**_**2**_ **O**_**3**_ **D**, **hSO**_**3**_		**5.53, 5.49**
A0A1B9F2X9	DITHS69_1544	Nucleic acid binding, OB‐fold, tRNA/helicase‐type		S_2_O_3_D	3.06	5.49
A0A1B9F585	DITHS69_0900	Adenylylsulfate reductase‐associated electron transfer protein QmoA	QmoA	S_2_O_3_D		5.49
A0A1B9F8V4	DITHS69_0589	Sulfurtransferase TusA/YTD cluster protein TusA	TusA	S_2_O_3_D	1.90	5.49
A0A1B9F720	DITHS69_0828	Sulfate adenylyltransferase	Sat	S_2_O_3_D		5.39
A0A1B9F4U6	DITHS69_0872	Uncharacterized protein/protein of unknown function		S_2_O_3_D		5.37
**A0A1B9F921**	**DITHS69_0444**	**Pyruvate:ferredoxin oxidoreductase (PFO), alpha subunit/Pyruvate synthase subunit PorA**	**PorA**	**S**_**2**_ **O**_**3**_ **D**, **hSO**_**3**_		**5.31**, **5.35**
**A0A1B9F742**	**DITHS69_0829**	**Phage protein**		**S**_**2**_ **O**_**3**_ **D**, **hSO**_**3**_		**5.27**, **5.40**
A0A1B9F5A6	DITHS69_0901	Adenylylsulfate reductase‐associated electron transfer protein QmoB	QmoB	S_2_O_3_D		5.26
A0A1B9F3F8	DITHS69_2096	DUF3373 domain‐containing protein		S_2_O_3_D		5.22
A0A1B9F3P7	DITHS69_1283	NAD‐dependent glyceraldehyde‐3‐phosphate dehydrogenase		S_2_O_3_D	1.42	5.21
A0A1B9F6K6	DITHS69_0026	Peptidoglycan‐associated lipoprotein	Pal	S_2_O_3_D	1.05	5.20
A0A1B9F6F6	DITHS69_0017	Formate—tetrahydrofolate ligase	Fhs	S_2_O_3_D		5.20
A0A1B9F312	DITHS69_0625	Fibronectin Type III domain protein		hSO_3_, S_2_O_3_D		5.75, 5.94
A0A1B9F4U6	DITHS69_0872	Uncharacterized protein/protein of unknown function		hSO_3_		5.74
A0A1B9F9F5	DITHS69_0271	NrfD‐type protein	NrfD	hSO_3_	−6.9	5.62
A0A1B9F937	DITHS69_0369	Chaperonin GroEL	GroL	hSO_3_	−1.35	5.51
A0A1B9F540	DITHS69_0904	Elongation factor Tu	TufB	hSO_3_	−1.43	5.51
A0A1B9F789	DITHS69_0801	ATP synthase subunit alpha		hSO_3_		5.48
A0A1B9F4Z1	DITHS69_0902	Adenylylsulfate reductase‐associated electron transfer protein QmoC	QmoC	hSO_3_	−2.09	5.47
A0A1B9F8N0	DITHS69_0586	Uncharacterized protein		hSO_3_	−0.96	5.46
A0A1B9F5K8	DITHS69_1003	Prepilin‐type N‐terminal cleavage/methylation domain‐containing protein		hSO_3_	−3.65	5.46
A0A1B9F478	DITHS69_2464	Amphi‐Trp domain‐containing protein		hSO_3_	−1.24	5.42
A0A1B9F5G6	DITHS69_0962	Large ribosomal subunit protein L16	RplP	hSO_3_	−1.82	5.42
A0A1B9F8G9	DITHS69_0590	Sulfur transport domain‐containing protein		hSO_3_	−2.75	5.41
A0A1B9F569	DITHS69_0986	Ketol‐acid reductoisomerase (NADP(+))		hSO_3_	−0.66	5.34

*Note:* Proteins that are among the 20 most abundant under both metabolisms (S_2_O_3_D, hSO_3_) are shown in bold. Proteins that are involved in sulfur metabolism are indicated in blue. Moreover, those proteins with a log_2_ fold change ≥ 0.58 (corresponding to a fold change ≥ 1.5) and an adjusted *p*‐value ≤ 0.05 were considered significantly upregulated under S_2_O_3_D, whereas those proteins with a log_2_ fold change ≤ −0.58 (also corresponding to a fold change ≥ 1.5) and an adjusted *p*‐value ≤ 0.05 were considered significantly downregulated under S_2_O_3_D (or equivalently upregulated under hSO_3_ condition). Abundance is expressed as log_10_q, where q = protein intensity (quantity).

**TABLE 2:** List of differentially abundant proteins involved in sulfur, iron and carbon metabolism during thiosulfate disproportionation (S_2_O_3_D).

ID protein	NCBI gene locus	Description	Symbol	Log_2_ FC (S_2_O_3_D/hSO_3_)	Adjusted *p*_met (S_2_O_3_D/hSO_3_)
***Sulfur metabolism‐related proteins***					
A0A1B9F2R9	DITHS69_2027	Molybdopterin oxidoreductase subunit with homologies to PhsB	PhsB*	**3.51**	0.001
A0A1B9F387	DITHS69_2028	Molybdopterin oxidoreductase subunit with homologies to PhsA	PhsA*	**2.45**	0.002
A0A1B9F9G3	DITHS69_0249	Universal stress protein		**2.29**	0.01
A0A1B9F4Z4	DITHS69_0898	Adenylylsulfate reductase beta‐subunit	AprB	2.20*, **	0.001
A0A1B9F563	DITHS69_0899	Adenylylsulfate reductase alpha‐subunit	AprA	1.42 *,**	0.004
A0A1B9F6N2	DITHS69_1147	Pyridine nucleotide‐disulfide oxidoreductase		2.06	0.002
A0A1B9F767	DITHS69_0181	NADH‐dependent butanol dehydrogenase		2.03	0.01
A0A1B9F8Z7	DITHS69_0396	Uncharacterized protein/DrsE domain‐containing protein		1.98	0.01
A0A1B9F8V4	DITHS69_0589	Sulfurtransferase TusA/YTD cluster protein TusA	TusA	1.90*	0.003
A0A1B9F8H6	DITHS69_1989	Transmembrane protein co‐occuring with sulfite exporter TauE/SafE		1.79	0.01
A0A1B9F8M2	DITHS69_1990	Probable membrane transporter protein/sulfite exporter TauE/SafE family protein	TauE/SafE	1.56	0.04
A0A1B9F9A5	DITHS69_0297	Dissimilatory sulfite reductase clustered protein DsrD	DsrD	1.28	0.03
A0A1B9F8W8	DITHS69_0436	Molybdopterin‐guanine dinucleotide biosynthesis protein MobB	MobB	1.24	0.04
A0A1B9F6E9	DITHS69_1071	Class III cytochrome *c* domain‐containing protein		1.20	0.002
A0A1B9F5X2	DITHS69_1690	Adenylate kinase	AK	1.10	0.048
A0A1B9F707	DITHS69_0859	Thioredoxin	Trx	1.09	0.03
‍A0A1B9F8Z0	DITHS69_0552	ATPase		0.88	0.03
***Iron metabolism‐related proteins***					
A0A1B9F5X6	DITHS69_0242	TonB‐dependent receptor/outer membrane receptor for ferrienterochelin and colicins FepA	TonB	**4.38**	0.01
***Nitrogen metabolism‐related proteins***					
A0A1B9F6A8	DITHS69_1138	Hydroxylamine reductase	Hcp	**2.81**	0.004
***Carbon metabolism‐related proteins***					
A0A1B9F8L2	DITHS69_1981	2‐oxoglutarate oxidoreductase, alpha subunit		**4.66**	0.001
A0A1B9F8F1	DITHS69_1988	Phosphoenolpyruvate synthase	PEP synthase	**3.40**	0.001
A0A1B9F798	DITHS69_0770	Malate dehydrogenase	mdh	1.57	0.03
A0A1B9F6S5	DITHS69_0084	Phosphoenolpyruvate carboxylase, archaeal	PEPC	0.71	0.03

*Note:* Proteins with a log_2_ fold change ≥ 0.58 (corresponding to a fold change ≥ 1.5) and an adjusted *p*‐value ≤ 0.05 were considered to have significantly increased abundance under S_2_O_3_D. Those proteins are among the 25 most upregulated under S_2_O_3_D are shown in bold, whereas the 20 most abundant proteins under S_2_O_3_D and hSO_3_ conditions are indicated by (*) and (**), respectively. The remaining proteins are listed in TableS2. For certain proteins, the description includes both UniProt and NCBI annotations, separated by a slash (/).

Enzymes involved in carbon metabolism, including the tricarboxylic acid cycle (TCA), gluconeogenesis, the Wood–Ljungdahl pathway (WLP) and the glyoxylate cycle, were detected under both S_2_O_3_D and hSO_3_ conditions. While enzymes of the TCA cycle and gluconeogenesis were more abundant under S_2_O_3_D (Table 2), those of the Wood–Ljungdahl and glyoxylate cycles were enriched under hSO_3_ conditions (Table 3). The enrichment of Wood–Ljungdahl (WL) pathway proteins under hSO_3_ conditions may reflect the greater availability of reducing power during hydrogenotrophic growth, whereas the higher abundance of enzymes involved in the tricarboxylic acid (TCA) cycle and gluconeogenesis under S_2_O_3_D conditions may indicate distinct anabolic or energetic constraints associated with sulfur disproportionation. The specific enzymes involved in these carbon metabolism pathways are detailed in the following sections.

**TABLE 3:** List of proteins related to sulfur, carbon, and iron metabolism, as well as other bioenergetic proteins, that were significantly more abundant under hSO_3_ conditions.

ID protein	NCBI gene locus	Description	Symbol	Log_2_ FC (S_2_O_3_D/hSO_3_)	Adjusted *p*_met (S_2_O_3_D/hSO_3_)
***Sulfur metabolism‐related proteins***					
A0A1B9F9K1	DITHS69_0267	4Fe‐4S protein		**−**0.99	0.02
A0A1B9F9F5	DITHS69_0271	Protein homologous to NrfD and PsrC	NrfD	−6.90**	**0.002**
A0A1B9F6J6	DITHS69_0039	Flagellar hook‐associated protein FlgL	FlgL	**−3.26**	**0.001**
A0A1B9F8G9	DITHS69_0590	Sulfur transport domain‐containing protein/YTD cluster protein YedE	YedE	**−**2.76	0.002
A0A1B9F883	DITHS69_1982	Universal stress protein		**−**1.78	0.01
A0A1B9F884	DITHS69_1984	Sulfur deprivation response regulator		**−**2.65	0.002
A0A1B9F7P4	DITHS69_0683	Molybdopterin oxidoreductase, 4Fe‐4S iron–sulfur subunit B		**−**1.57	0.02
A0A1B9F7L3	DITHS69_0684	Molybdopterin oxidoreductase, Ttr homologue		**−**2.63	0.001
A0A1B9F6D9	DITHS69_1081	Porin with homoloy with the phosphate‐selective porin OprOP		**−**2.27	0.02
A0A1B9F6B7	DITHS69_1082	Tetrathionate reductase subunit B	TtrB	**−**1.40	0.03
A0A1B9F6F3	DITHS69_1084	Tetrathionate reductase subunit A	TtrA	**−**1.89	0.005
A0A1B9F4Z1	DITHS69_0902	Adenylylsulfate reductase‐associated electron transfer protein QmoC	QmoC	**−**2.09**	0.003
A0A1B9F4R4	DITHS69_1420	Molybdopterin oxidoreductase subunit A	MolyA	**−**2.09	0.002
‍A0A1B9F6R1	DITHS69_0111	Sulfur transport domain‐containing protein /YeeE/YedE family sulfur transport domain‐containing protein	YeeE/YedE	**−**2.08	0.01
A0A1B9F9D1	DITHS69_0329	Sulfite reduction‐associated complex DsrMKJOP protein DsrP (HmeB)	DsrP	**−**1.72	0.002
A0A1B9F996	DITHS69_0331	Sulfite reduction‐associated complex DsrMKJOP multiheme protein DsrJ (HmeF)	DsrJ	**−**1.85	0.02
A0A1B9F9K7	DITHS69_0332	Sulfite reduction‐associated complex DsrMKJOP protein DsrK (HmeD)	DsrK	**−**1.40	0.01
A0A1B9F982	DITHS69_0333	Sulfite reduction‐associated complex DsrMKJOP protein DsrM (HmeC)	DsrM	**−**2.07	0.003
A0A1B9F2L0	DITHS69_2230	Flagellin		**−**1.31	0.046
A0A1B9F960	DITHS69_0354	Nif‐specific regulatory protein		**−**1.24	0.004
A0A1B9F8F0	DITHS69_1920	Flagellar protein FliL	FliL	**−**0.86	0.01
A0A1B9F5L0	DITHS69_1615	CoB—CoM heterodisulfide reductase, subunit C	HdrC	**−**2.74	0.005
A0A1B9F5K9	DITHS69_1618	CoB—CoM heterodisulfide reductase, subunit A	HdrA	**−**0.84	0.02
A0A1B9F5U0	DITHS69_1621	Methyl‐viologen‐reducing hydrogenase subunit gamma	Mvhγ	**−**1.85	0.001
A0A1B9F5X0	DITHS69_1622	Methyl‐viologen‐reducing hydrogenase, ubunit alpha	Mvhα	**−**1.17	0.001
***Iron metabolism‐related proteins***					
A0A1B9F5Z7	DITHS69_1252	TonB‐dependent receptor		**−3.38**	**0.03**
A0A1B9F9G0	DITHS69_0242	TonB‐dependent receptor		**−**1.12	0.002
A0A1B9F6V9	DITHS69_0164	TonB‐dependent receptor		**−**0.92	0.02
***Carbon metabolism‐related proteins***					
A0A1B9F6D3	DITHS69_1103	Re face‐specific citrate synthase		**−**1.40	0.01
A0A1B9F5X9	DITHS69_1234	Glycolate oxidase iron–sulfur subunit	GlcF	**−**1.21	0.03
A0A1B9F8G0	DITHS69_0575	CO dehydrogenase accessory protein CooC (Nickel insertion)		**−**0.90	0.04
A0A1B9F5R2	DITHS69_1706	Glycolate oxidase, subunit GlcD	GlcD	**−**0.82	0.01
A0A1B9F6X0	DITHS69_2496	Protein with homologies to formate dehydrogenase subunit alpha, but with size not in line		**−**0.99	0.03
***Hydrogen metabolism***					
A0A1B9F8Z4	DITHS69_0472	[NiFe] hydrogenase metallocenter assembly protein HypC/hydrogenase expression/formation protein HypC	HypC	**−**1.70	0.03
A0A1B9F8U2	DITHS69_0470	[NiFe] hydrogenase metallocenter assembly protein HypE/hydrogenase maturation protein, carbamoyl dehydratase	HypE	**−**1.14	0.01
***Other bioenergetic proteins***					
A0A1B9F3T6	DITHS69_2242	Multiheme cytochrome *c*		**−**2.18	0.007
A0A1B9F9S3	DITHS69_0259	Class III cytochrome *c* family protein		**−**2.04	0.003
A0A1B9F3W6	DITHS69_2265	NADH‐quinone oxidoreductase subunit N	NuoN	**−**2.48	0.005
A0A1B9F431	DITHS69_2266	NADH–ubiquinone oxidoreductase chain M	NuoM	**−**2.91	0.002
A0A1B9F465	DITHS69_2267	NADH–ubiquinone oxidoreductase chain L	NuoL	**−3.26**	**0.001**
A0A1B9F3V3	DITHS69_2270	NADH‐quinone oxidoreductase subunit I	NuoI	**−**1.83	0.004
A0A1B9F3W4	DITHS69_2271	NADH‐quinone oxidoreductase subunit H	NuoH	**−**2.49	0.003
A0A1B9F3V1	DITHS69_2272	NADH‐quinone oxidoreductase subunit D	NuoD	**−**2.09	0.005
A0A1B9F3X3	DITHS69_2273	NADH‐quinone oxidoreductase subunit C	NuoC	**−**2.18	0.007
A0A1B9F4F5	DITHS69_2274	NADH‐quinone oxidoreductase subunit B	NuoB	**−**2.03	0.02
A0A1B9F4A3	DITHS69_1269	Cytochrome *c*‐type biogenesis protein Ccs1/ResB		**−**1.41	0.01
A0A1B9F3M3	DITHS69_1305	Cytochrome *c*		**−**0.70	0.007
‍A0A1B9F5N5	DITHS69_1675	Menaquinone biosynthesis C methyltransferase UbiE		**−**1.41	0.01
A0A1B9F5T8	DITHS69_1716	Cytochrome *c* family protein		**−**0.90	0.009
A0A1B9F3F7	DITHS69_2139	Cytochrome *c* family protein		**−**1.10	0.003
A0A1B9F754	DITHS69_0799	ATP synthase subunit b	atpF	**−**1.85	0.006
A0A1B9F789	DITHS69_0801	ATP synthase subunit alpha	atpA	**−**1.17**	0.03
A0A1B9F7F7	DITHS69_0804	ATP synthase subunit beta	atpD	**−**1.44	0.008
A0A1B9F7I4	DITHS69_0818	ATP synthase subunit a	atpB	**−**1.62	0.003
A0A1B9F7A1	DITHS69_0822	ATP synthase protein I		**−**0.85	0.04

*Note:* Proteins with a log_2_ fold change ≤ −0.58 (corresponding to a fold change ≥ 1.5) and an adjusted *p*‐value ≤ 0.05 were considered to have significantly increased abundance under hSO_3_ condition. Those proteins among the 25 most upregulated under hSO_3_ are shown in bold, whereas the 20 most abundant proteins under S_2_O_3_D and hSO_3_ conditions are indicated by (*) and (**), respectively. The remaining proteins are listed in TableS2. For certain proteins, the description includes both UniProt and NCBI annotations, separated by a slash (/).

The broader set of proteins showing increased abundance under hSO_3_ is consistent with the metabolic product profiles, suggesting the possible simultaneous occurrence of sulfite respiration and disproportionation (Figure 2B).

#### 3.2.1 Thiosulfate Disproportionation

**FIGURE 3:**
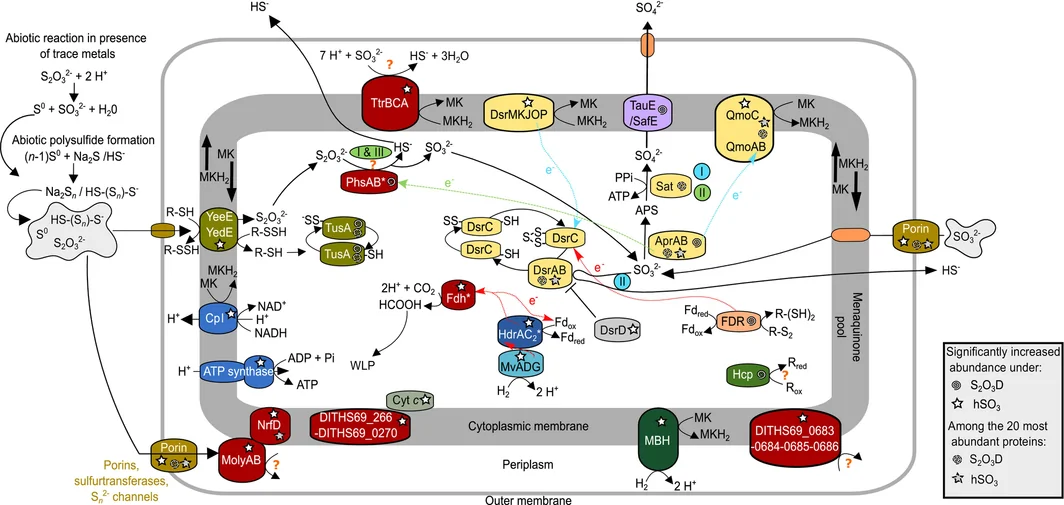
Diagram presenting (i) proposed hypothetical metabolic models for thiosulfate (green circles) and sulfite disproportionation (blue circles) in strain S69ᵀ, with reaction steps indicated by numbers, and (ii) enzymes showing increased abundance under hSO_3_ conditions. Proteins showing significantly increased abundance under S_2_O_3_D conditions are marked with a spiral symbol, whereas proteins showing significantly increased abundance under hSO_3_ are marked with a star symbol. Reactions not highlighted by the proteomic results are based on the genetic potential and the published functions of the corresponding enzymes. Enzyme names are listed in Tables 1, 2, 3. Enzymes in red boxes represent unknown complexes of subunits, some of which share homology with known subunits. Question marks indicate newly hypothesized reactions or novel reaction directions. * indicates that these enzymes differ from the canonical forms. Additional details are provided in the main text.

Thiosulfate import across the outer membrane may involve outer membrane sulfurtransferases or porins, which have yet to be identified. Once in the periplasm, transport of thiosulfate (and potentially polysulfides) across the inner membrane could be mediated by one of the two YeeE/YedE family proteins, which contain a sulfur transport domain and were detected under S_2_O_3_D conditions. One of this protein (A0A1B9F6R1/DITHS69_0111) is not encoded by the *YTD* gene cluster, while the other one (A0A1B9F8G9/DITHS69_0590) is part of the *YTD* cluster. In the cytoplasm, thiosulfate could be reductively cleaved into sulfite and sulfide by a thiosulfate reductase‐like complex, homologous to PhsAB (A0A1B9F387/DITHS69_2028; A0A1B9F2R9/DITHS69_2027) (Figure 3). This complex, lacking the PhsC subunit, was predicted to be cytoplasmic due to the absence of the *tat* leader sequence, which is typically involved in the addressing of molybdoenzyme fully folded monomeric or dimeric subunits across the cytoplasmic membrane (Rothery et al. 2008; Supplementary Table S9). Its two subunits were among the proteins showing the greatest increases in abundance under S_2_O_3_D conditions, with relative abundances of 2.45 and 3.51, respectively, suggesting a functional role in thiosulfate metabolism (Figure 2C, Table 2). The PhsAB‐like complex exhibited the highest protein sequence identity (> 60%) with Fe‐S and molybdenum subunits from other sulfur‐disproportionating bacteria, notably those from *Desulfolithobacter dissulfuricans* (Hashimoto et al. 2022; Supplementary Table S9). It has been proposed that this enzyme catalyses the initial step of the thiosulfate disproportionation pathway by reductive cleavage of thiosulfate into sulfite and sulfide in certain taxa (Florentino et al. 2019; Dahl 2020; Hashimoto et al. 2022). While this enzyme may not play a major role in *Desulfolithobacter dissulfuricans* strain GF1^T^ under thiosulfate‐disproportionating conditions (Hashimoto et al. 2022), the opposite appeared to be true for the strain studied here. It would be of great interest to characterise the physiological function of this PhsAB‐like enzyme in future studies and investigate its potential membrane association, given its apparent central role in S_2_O_3_D metabolism in strain S69^T^.

Then, the oxidative branch of thiosulfate disproportionation likely proceeded via a reversal of the canonical sulfate reduction pathway, involving the highly abundant Apr and Sat enzymes, as suggested previously (Hashimoto et al. 2022; Sorokin et al. 2025). These enzymes would enable the conversion of sulfite to sulfate, likely coupled to ATP production through substrate‐level phosphorylation (Krämer and Cypionka 1989). The two electrons released in the sulfite to sulfate oxidation step could then be used by the PhsAB‐like to reduce thiosulfate, generating sulfide and sulfite (Figure 3). This disconnection from the membranous electrons, usually coming from quinones, would explain the absence of a membranous subunit in this PhsAB. This hypothetical model, based on genomic and proteomic data, partially aligns with findings reported for *Desulfolitobacter dissulfuricans* (analysed for thiosulfate disproportionation) and *Desulfurivibrio dismutans* (studied for elemental sulfur disproportionation) (Hashimoto et al. 2022; Sorokin et al. 2025), the only two sulfur‐disproportionating *Desulfobacterota* strains characterised to date via proteomics for their sulfur compound disproportionation pathways. It is important to note, however, that in these three examples, AprAB and Sat were predicted by HMM profiles to be of the reductive type, whereas the hypothetical models propose the opposite directionality. However, the initial reaction of sulfur compound disproportionation and the reductive branch may vary, at least in part, among these three species and depending on the initial sulfur substrate used for disproportionation. It was shown that *D. dismutans* exhibited increased abundance of a polysulfide reductase PsrABC‐like upon elemental sulfur disproportionation, leading to sulfide production (Sorokin et al. 2025). In contrast, in *D. dissulfuricans*, under thiosulfate disproportionation conditions, a close homologue of membranous tetrathionate reductase (TtrBCA) (Supplementary Table S9) showed increased abundance, and one subunit A (out of two) of a soluble form of a close homologue to thiosulfate reductase PhsAB was slightly more abundant under S_2_O_3_D conditions than under sulfate‐reducing conditions (Hashimoto et al. 2022). The authors acknowledge that no single scenario could be definitively validated from these proteomic experiments. Nevertheless, one proposed hypothesis was that tetrathionate, produced from thiosulfate by a tetrathionate reductase‐like TtrBCA, may be consumed through a reaction with TusA and DsrE. This reaction would involve sulfur transfer via sulfane sulfur intermediates to the Dsr system, potentially leading to reduction to sulfide. However, the involvement of a PhsAB‐like enzyme in the reductive cleavage of thiosulfate was not excluded either. Based on careful examination of all the markers known from the biochemical representative of tetrathionate reductase (including number of transmembrane helices, subunit gene length), *D. thermophilus* also encodes a close homologue of tetrathionate reductase *ttrBCA*. It also encodes a YTD gene cluster that includes a DsrEFH Type II. Notably, TusA was significantly more abundant under thiosulfate‐disproportionating (S_2_O_3_D) conditions. It would be valuable in future work to investigate whether *D. thermophilus* produces tetrathionate under S_2_O_3_D conditions and whether the pathway proposed for *D. dissulfuricans* might also operate in *D. thermophilus* . The elevated abundance of TusA might alternatively be explained by the presence of polysulfides, which could form abiotically under the experimental conditions through reactions between sulfide and elemental sulfur in the presence of trace iron hydroxides (Figure 3). These polysulfides might then participate in TusA‐mediated sulfur transfer reactions involving sulfane sulfur intermediates, though this remains speculative.

To date, no experimental evidence supports the presence of dedicated H_2_S export systems in *Desulfobacterota*. Given its lipophilic nature, hydrogen sulfide (H_2_S) is generally assumed to leave the cell by passive diffusion across lipid bilayers, a mechanism supported by studies reporting its high membrane permeability and negligible resistance to diffusion (Mathai et al. 2009; Cuevasanta et al. 2012; Riahi and Rowley 2014; Kimura 2015). However, since HS^−^ predominates at physiological pH, it may either be converted into H_2_S to enable passive diffusion or potentially be released through membrane channels (Kimura 2015).

On the other hand, sulfate export is generally not mediated by specific active transporters; instead, sulfate likely moves across membranes via facilitated diffusion or through ion exchange systems (antiporters) capable of handling multiple anions, as supported by studies on bacterial sulfate transport mechanisms (Piłsyk and Paszewski 2009). A protein from the TauE/SafE family of sulfite exporters (A0A1B9F8M2/DITHS69_1990) and an associated transmembrane protein (A0A1B9F8H6/DITHS69_1989) were significantly more abundant under thiosulfate disproportionation (1.56 Log_2_ FC), suggesting a potential role in regulating sulfite flux or the export of other sulfur compounds during this process (Figures 2C, 3). However, it is important to note that sulfite, a putative intermediate of the reaction, was not detected during incubation (detection threshold of the method: 1 μM).

In addition, a TonB‐dependent receptor (A0A1B9F5X6/DITHS69_0242) associated with iron metabolism was also detected at higher abundance under S_2_O_3_D conditions compared to hSO_3_ conditions. The increased abundance of these proteins (ATPase and TonB‐dependent receptor) might also be induced by residual traces of Fe(OH)_3_ in the inoculum. A hydroxylamine reductase (Hcp), in synteny with a pyridine nucleotide‐disulfide oxidoreductase that also showed increased abundance under S_2_O_3_D conditions, was likewise detected at higher abundance despite the absence of hydroxylamine in the medium (Table 2). This activation might be due to global regulation or cross‐regulation, or to the fact that this enzyme is involved in the reduction process of a non‐nitrogenous compound, as already documented elsewhere (Xu et al. 2024).

Furthermore, malate dehydrogenase (A0A1B9F798/DITHS69_0770), the alpha subunit of 2‐oxoglutarate oxidoreductase (A0A1B9F8L2/DITHS69_1981), PEP synthase (A0A1B9F8F1/DITHS69_1161), and PEP carboxylase (A0A1B9F6S5/DITHS69_0084) were more abundant under S_2_O_3_D, suggesting active operation of the TCA cycle and gluconeogenesis (Table 2).

#### 3.2.2 Reactions at Work in Hydrogenotrophic Sulfite‐Containing Cultures

As several metabolic processes probably co‐occurred under hydrogenotrophic sulfite‐containing (hSO_3_) condition, it was more difficult to draw clear conclusions. Proteins that were significantly more abundant under these conditions are listed in Table 3. Consistent with the sulfur products observed during metabolism (Figure 1B), these proteins further support the potential co‐occurrence of sulfite/thiosulfate reduction and disproportionation.

Sulfite could enter the cell through specific porins, ionic channels or transporters, or through ABC transporters or symporters. A protein homologous to the phosphate‐selective porin OprOP (A0A1B9F6D9/DITHS69_1081), from which the gene is located in synteny with the *ttrBCA*‐homologue gene cluster, was more abundant under hSO_3_ conditions (Table 3). This may indicate its potential involvement in the import of as‐yet‐undetermined compounds, possibly sulfur compounds; however, this hypothesis requires experimental validation. Alternatively, sulfite could be converted by one of the identified oxidoreductases described below into a sulfur compound that could then be transported by YeeE/YedE family proteins (A0A1B9F6R1/DITHS69_0111 and A0A1B9F8G9/DITHS69_0590). This would explain why both YeeE/YedE family proteins showed increased abundance under hSO_3_ conditions (−2.08 and −2.76 Log_2_ FC S_2_O_3_D/hSO_3_, Figure 2C, Table 3).

Among the proteins showing the greatest increases in abundance under hSO_3_ conditions, several uncharacterized proteins potentially involved in sulfite reduction and/or sulfite disproportionation were identified. These included an enzyme containing a protein module homologous to NrfD (A0A1B9F9F5/DITHS69_0271; −6.90 Log_2_ FC, Figure 2C, Table 3; Supplementary Table S9). NrfD is a membranous protein devoid of any cofactor that takes part in highly diverse complexes, among them the Nrf complex and many molybdenum enzymes (Rothery et al. 2008; Duarte et al. 2021). Genome examination did not identify any potential genes coding for other subunits of well‐known complexes in the vicinity of the NrfD gene but identified genes (DITHS69_0268‐0271 and DITHS69_0272) with homology to transcriptional regulators on the same strand as NrfD, and one tetraheme cytochrome *c* subunit (A0A1B9F9D7/DITHS69_0266) and one Fe‐S subunit (DITHS69_0267) on the opposite strand.

Although it exhibited a more moderate difference in abundance (−2.09 Log_2_ FC), a molybdopterin oxidoreductase subunit A (MolyA, A0A1B9F4R4/DITHS69_1420) was also detected under hSO_3_ conditions, suggesting a potential role in sulfite reduction or disproportionation (Figure 2C, Table 3). This protein belongs to the Moly protein cluster proposed to be involved in sulfur compound disproportionation based on comparative genomics (Allioux et al. 2020). Additionally, subunits of another molybdopterin oxidoreductase (A0A1B9F7L3/DITHS69_0684, A0A1B9F7P4/DITHS69_0683), likely representing a novel class of Ttr homologues lacking the TtrC subunit (Supplementary Table S9), were more abundant under hSO_3_ conditions. The gene cluster, on the other hand, contains two genes. The first one encodes an 8‐transmembrane helices protein (A0A1B9F7M8/DITHS69_0685) while the second encodes a 10‐transmembrane helices protein (A0A1B9F7I2/DITS69_0686). This gene cluster would therefore represent a novel type of membranous molybdopterin enzyme. Moreover, the subunits B and A (A0A1B9F6B7/DITHS69_1082, A0A1B9F6F3/DITHS69_1084) of a close homologue to TtrBCA (which exhibited the expected sequence size and motifs) were detected at higher abundances (−1.89 and −1.40 Log_2_ FC, respectively; Figure 2C, Table 3). TtrC (A0A1B9F6D1/DITHS69_1083) (which also presents all the sequence motifs of a true TtrC) may not have been detected due to the inherent difficulty in detecting membrane proteins. Although TtrBCA has been shown to be involved in sulfur disproportionation in certain taxa (Hashimoto et al. 2022), and proposed for others (Muramatsu et al. 2020), its specific role under the conditions tested in this study remains unresolved. A function in tetrathionate reduction appears unlikely because tetrathionate was not provided in the medium, and its formation was considered unlikely and was not monitored. Given the culture conditions, it can be speculated that this enzyme is involved in sulfite reduction. To date, TtrBCA homologues have only been demonstrated to catalyse tetrathionate reduction (Hensel et al. 1999; Degré et al. 2026) or arsenate reduction (Haja et al. 2020; Muramatsu et al. 2020), but their activity towards sulfite has not been tested. Thermodynamically, the reduction of sulfite (SO_3_^2−^/HS^−^, E°′ = −116 mV; Thauer et al. 1977) is unfavourable when coupled to menaquinone oxidation (E°′ = −74 mV; Thauer et al. 1977), unlike the reduction of tetrathionate (E°′ = +198 mV; Kurth et al. 2015) or arsenate (E°′ = +139 mV; Vink 1996). However, it should be noted that the reduction of sulfite from the oxidation of menaquinone is the physiological function of the SirACD membrane enzyme (Shirodkar et al. 2011) as is the reduction of thiosulfate (Em = −402 mV; Thauer et al. 1977) from the oxidation of menaquinone, which is the physiological function of the PhsABC membrane enzyme (Stoffels et al. 2012). Structurally, sulfite and arsenate share similarities and are smaller than tetrathionate, making their binding to the enzyme's active site plausible. To resolve this uncertainty, further enzymatic activity assays and/or mutant construction, coupled with sensitive sulfur speciation analyses, will be required to determine the enzyme's function and confirm or refute its hypothetical role in sulfite reduction. Other proteins that were relatively more abundant under hSO_3_ conditions included several components of the sulfite reduction‐associated DsrMKJOP complex (HmeCFBD) (Figure 3, Table 3). This membrane‐bound complex typically transfers electrons from the menaquinone pool to the cytoplasmic protein DsrC, which in turn donates them to the catalytic sulfite reductase DsrAB (Santos et al. 2015). Notably, a C‐methyltransferase UbiE/MenG (A0A1B9F5N5/DITHS69_1675) required for the final methylation step in menaquinone biosynthesis was significantly more abundant under hSO_3_ (−1.41 Log_2_ FC), suggesting a key role in maintaining menaquinone‐dependent electron transport in strain S69^T^. Additional iron‐related TonB‐dependent receptor proteins, related to those detected at higher abundance under S_2_O_3_D conditions, were also identified under hSO_3_ conditions (A0A1B9F5Z7/DITHS69_1252, A0A1B9F9G0/DITHS69_0242, A0A1B9F6V9/DITHS69_0164) (Tables 2 and 3). Based on the proteomic data, on the genomic content of the strain and of the described functions of the encoded enzymes and abundantly produced proteins, one can speculate that the reductive branch might involve DsrABC with electrons supplied either by the DsrMKJOP complex or another complex, and that the oxidative branch of sulfite disproportionation might involve the AprAB and Sat enzymes with electrons being funnelled back to the menaquinone pool via QmoABC (Figure 3).

Overall, a greater number of membrane‐bound enzymes involved in redox processes/electron transport and proton motive force generation showed increased abundance under hydrogenotrophic sulfite conditions (hSO_3_) than under thiosulfate disproportionation (S_2_O_3_D) (Figure 3). As shown in Figure 3 (and listed in Table 3), several subunits of ATP synthase, as well as respiratory chain Complex I (*nuo* genes) (DITHS69_2265‐DITHS69_2274) were significantly more abundant under hSO_3_ conditions. Moreover, six cytochrome *c* proteins also showed increased abundance under the hydrogenotrophic sulfite‐reducing (hSO_3_) condition (Table 3). The increased abundance of these proteins is consistent with enhanced respiratory activity and may indicate the involvement of an additional energy conservation mechanism, potentially driven by the greater availability of Gibbs free energy. These findings are consistent with the previously described physiological capacities of this strain, which include the ability to perform anaerobic respirations (Slobodkin et al. 2013). Anaerobic disproportionation of inorganic sulfur compounds is often termed inorganic fermentation (Finster 2008; Slobodkin and Slobodkina 2019), since it occurs under strictly anaerobic conditions and, based on current understanding, relies on substrate‐level phosphorylation for ATP production rather than involving a respiratory chain and proton motive force. However, since the full pathway of sulfur compound disproportionation remains only partially understood, the potential involvement of both inorganic fermentation and oxidative phosphorylation in sulfur compound disproportionation cannot be ruled out. Similarly, in cases where sulfite respiration and sulfite disproportionation co‐occur, it is expected that both energy conservation mechanisms—oxidative phosphorylation and substrate‐level phosphorylation—are involved.

Among the other proteins showing increased abundance under hSO_3_ conditions, two subunits (α and γ) (A0A1B9F5X0/DITHS69_1622 and A0A1B9F5U0/DITHS69_1621) of the methyl viologen‐reducing hydrogenase MvhAGD (DITHS69_1619 to DITHS69_1622) and two subunits (α and γ) (A0A1B9F5K9/DITHS69_1618 and A0A1B9F5L0/DITHS69_1615) of a heterodisulfide reductase‐like complex HdrAC_2_ (DITHS69_1615 to DITHS69_1618) (Table 3, Supplementary Table S10) were detected at higher abundance under hSO_3_ conditions (Appel et al. 2021). MvhAGD likely oxidises H_2_ to generate electrons, which were likely transferred to HdrA and bifurcated to reduce a ferredoxin serving as a low‐potential electron acceptor and possibly to a putative formate dehydrogenase (A0A1B9F6X0/DITHS69_2496; A0A1B9F3T2/DITHS69_2497), which showed increased abundance under hSO_3_ and may serve as a high‐potential electron acceptor (Appel et al. 2021). Some of the electrons may be funnelled to this putative formate dehydrogenase, potentially supporting autotrophic CO_2_ fixation via the Wood–Ljungdahl pathway (WLP; see Supporting Information for enzyme details and locus‐tags), as previously proposed (Yvenou et al. 2022; Sorokin et al. 2025; Yvenou et al., submitted) or channelled towards the formation of organic acids, including formate (Figure 3). The production of formate was not examined in this experiment, but has been observed upon growth of strain S69^T^ by S^0^ disproportionation (Yvenou et al., submitted). H_2_ oxidation under hSO_3_ conditions may also involve a membrane‐anchored periplasmic hydrogenase (referenced as MBH Membrane‐Bound Hydrogenase in Figure 3). This enzyme is encoded by the *hyp* gene cluster (DITHS69_0467‐DITHS69_0476), several subunits and maturation proteins were detected at higher abundance under these conditions (Table 3). This suggests the formation of functional [NiFe] hydrogenases capable of transferring electrons to the quinone pool in the membrane during sulfite respiration.

Proteins related to flagellar systems, particularly FlgL, as well as flagellin and FliL, showed increased abundance under hSO_3_ conditions. A complete set of carbon metabolism enzymes was also detected under the hydrogenotrophic sulfite‐containing (hSO_3_) conditions. Subunits of glycolate oxidase (A0A1B9F5X9/DITHS69_1234, A0A1B9F5R2/DITHS69_1706) and citrate synthase (A0A1B9F6D3/DITHS69_1103) were significantly more abundant under hSO_3_ conditions. This suggests a stronger connection between the reductive acetyl‐CoA (Wood–Ljungdahl) pathway and central metabolism, as well as increased involvement of the glyoxylate cycle. The enrichment of glyoxylate cycle enzymes may be linked to WLP activation, which produces acetyl‐CoA to replenish the TCA cycle or generate gluconeogenesis precursors (Petushkova et al. 2019). Alternatively, it could reflect glycolate synthesis, as previously observed under S^0^‐disproportionation conditions in strain S69^T^ (Yvenou et al., submitted).

#### 3.2.3 Provisional Model of Metabolic Reactions Occurring Under Both Conditions

Figure 3 presents a provisional model, based on genomic and proteomic data, describing thiosulfate disproportionation and sulfite disproportionation in strain S69^T^. It also highlights all enzymes that showed increased abundance under hSO_3_ conditions.

Overall, for thiosulfate disproportionation, thiosulfate would likely enter the cell through specific porins or transporters and could be subsequently transported into the cytoplasm via YedE or another transporter. Once in the cytoplasm, thiosulfate could be reductively cleaved by a cytoplasmic PhsAB‐like enzyme into sulfide as the final product, and sulfite, which could then enter the oxidative branch. In the oxidative branch, sulfite could be oxidised to sulfate through the sequential actions of AprAB and Sat, with electrons being funnelled back to PhsAB, and sulfate may subsequently be exported from the cell via transporters of the TauE/SafE family (Figure 3). In addition, Hdr may transfer electrons to putative formate dehydrogenase (Fdh) (Appel et al. 2021), facilitating formate production, and to the glyoxylate pathway facilitating glycolate production; this tight coupling between the sulfur and carbon cycles could provide an energetic advantage under the stringent conditions where sulfur disproportionation occurs.

The results obtained under hydrogenotrophic conditions with sulfite (hSO_3_) were more challenging to interpret, as sulfite respiration and disproportionation likely co‐occurred. Sulfite may be imported into the cytoplasm via porins, ionic channels, transporters, or symporters. Under these conditions, a membrane‐bound hydrogenase and a cytoplasmic methyl viologen‐reducing hydrogenase (MvhAGD), associated with the heterodisulfide reductase complex (HdrAC_2_), were very likely involved in hydrogen oxidation, with electrons likely transferred to the diffusible quinone pool in the membrane for the first, and to central metabolism or organic acid formation for the second. Molybdopterin‐containing oxidoreductase subunits—including a complex containing subunits homologous to TtrA and B, a true genetic homologue of TtrBCA and another complex called MolyAB—were also detected at increased abundances. However, deciphering their precise roles from proteomic and genomic data alone was not possible. Under these conditions, an additional energy conservation mechanism may involve the membrane‐bound Complex I of the respiratory chain (Nuo complex; NADH:quinone oxidoreductase), contributing to overall redox balance and energy efficiency. Moreover, ATP generation under these conditions where respiration and disproportionation co‐occur was likely supported by both substrate‐level phosphorylation involving sulfate adenylyltransferase (Sat) and oxidative phosphorylation through ATP synthase. The reductive branch of sulfite disproportionation could involve DsrABC for reduction to sulfide and the oxidative branch could involve AprAB and Sat enzymes for the oxidation of sulfite to sulfate (despite their predicted reductive directionality based on sequence analysis).

These observations reinforce growing evidence that bacteria apparently recruit enzymes traditionally associated with other sulfur cycle processes to carry out sulfur disproportionation, in combination with novel bioenergetic module combinations that form new enzymes.

## 4 Conclusion

In this study, proteomic data were generated for strain S69ᵀ, isolated from a deep‐sea hydrothermal vent environment, during growth by thiosulfate disproportionation and in the presence of sulfite and hydrogen. These results provided new insights into the energy conservation mechanisms in this strain. By combining substrates/product and growth monitoring with in‐depth proteomic analysis, the growth, metabolic profile, and proteome of the strain were characterised under two different nutritional conditions. The strain was able to grow via thiosulfate disproportionation (N_2_/CO_2_) and showed enhanced growth in the presence of sulfite, likely through a combination of sulfite respiration and disproportionation (H_2_/CO_2_). This simultaneous use of two metabolisms under hSO_3_ conditions was unexpected and shed light on the metabolic flexibility of this strain. It might indicate that the disproportionation metabolism of sulfur compounds was still switched on in this strain, even if further experiments will be necessary to confirm it. This result also supports the idea proposed by other authors (Wasmund et al. 2017; Yvenou et al. 2022) that sulfur disproportionation may serve as a survival strategy or an alternative metabolic pathway under energy‐limited or geochemically unfavourable conditions, particularly when only a single sulfur species is available, or when conventional electron donors and acceptors are scarce. Although further biochemical studies such as enzymatic activities or mutant studies are required to validate this proteomics‐based model, the proposed pathways offer valuable insights into the metabolic mechanisms underlying sulfur disproportionation in strain S69^T^—the fourth organism characterised at the protein level for this metabolism. These results indicated that the oxidative branch of sulfur compound disproportionation was likely mediated by the activity of enzymes of the dissimilatory sulfate reduction pathway (AprAB and Sat) operating in reverse direction in the three *Desulfobacterota* genera described so far. These results also indicated that the reductive branch of sulfur compound disproportionation may be catalysed by either DsrABC, PhsAB or PsrABC in the three *Desulfobacterota* strains whose proteomes have been analysed. Additionally, this process may involve various molybdopterin oxidoreductases, including PhsAB‐like, TtrBCA, and MolyAB. These enzymes represent novel combinations of bioenergetic protein modules or are recruited from other metabolic reactions within the sulfur cycle, depending on the genus. Together, these findings highlight the mosaic nature of sulfur disproportionation pathways and enzymes, a hallmark of bioenergetic routes, likened by others to ‘Lego blocks’ or ‘redox kits’ (Padalko et al. 2025).

Future studies should focus on determining the biochemical and biophysical properties of the diverse molybdopterin oxidoreductases—including PhsAB‐like, TtrBCA, MolyAB, and DITHS69_0683‐0686—all of which were detected at higher abundances under sulfur compound disproportionation or reduction conditions. Elucidating their precise roles will be essential for a deeper understanding of sulfur metabolism. It would also be useful to conduct incubations with radiolabeled sulfur compounds to track as many sulfur compounds (e.g., sulfite, thiosulfate, polysulfides, tetrathionate, and elemental sulfur) as possible in sulfur disproportionation pathways within *Desulfobacterota*. This approach could particularly help confirm whether sulfite was a metabolic intermediate in the reaction, as suspected. It would also be important to compare proteomes generated under various disproportionation conditions with different sulfur compounds (e.g., sulfite, thiosulfate, and elemental sulfur) to assess similar energetic states and redox balances, especially since some central sulfur metabolism enzymes may be constitutively expressed at high abundance, regardless of their physiological role. Combining these analyses with transcriptomic data could help identify putative regulators and address the underrepresentation generally observed of membrane proteins in proteomic studies. Furthermore, future studies should explore additional metabolic pathways, such as S^0^ oxidation coupled with dissimilatory nitrate reduction to ammonium (DNRA), and Fe(III) reduction, which will be essential to complete the metabolic profile of this ecologically relevant organism.

## Author Contributions

**Barbara Schoepp‐Cothenet:** validation, writing – review and editing. **Sébastien Laurent:** investigation, writing – review and editing, methodology. **Lukas V. F. Novák:** investigation, writing – review and editing, validation, supervision, data curation. **Xavier Philippon:** investigation, methodology, writing – review and editing. **Céline Henry:** funding acquisition, writing – review and editing, validation, supervision, resources, project administration, methodology. **Karine Alain:** conceptualization, investigation, funding acquisition, writing – original draft, validation, writing – review and editing, visualization, methodology, formal analysis, project administration, resources, supervision, data curation. **Marie Hemon:** methodology, writing – review and editing. **Carine Rodrigues‐Machado:** methodology, writing – review and editing, supervision. **Marilina Fernandez:** investigation, writing – original draft, methodology, validation, visualization, writing – review and editing, formal analysis, data curation, supervision, conceptualization.

## Funding

This work was supported by French National Research Agency, ANR‐22‐CE02‐0001.

## Ethics Statement

This manuscript is an original work complying with standard scientific ethical practices.

## Conflicts of Interest

The authors declare no conflicts of interest.

## Supporting information


**Data S1:** Supporting Information.
**Figure S1:** Growth of strain S69^T^ via disproportionation of thiosulfate (A), and under hydrogenotrophic sulfite‐containing (hSO_3_) conditions (B). Full growth curves covering all growth phases are shown in grey, while the growth kinetics for proteomic sampling are shown in black. Proteins were extracted from the last black point on each curve, corresponding to the mid‐exponential growth phase. Temporal changes in thiosulfate, sulfate, sulfide, and sulfite concentrations—after subtracting the corresponding abiotic controls—in cultures of strain S69ᵀ grown under thiosulfate disproportionation (S_2_O_3_D) conditions (C) and in hydrogenotrophic sulfite‐containing medium (hSO_3_).
**Figure S2:** Results of peptide *m/z* intensity normalisation using a median‐based method: (A) intensity‐based violin plot, (B) principal component analysis (PCA) and (C) intensity distribution plot and of the different biological replicates. References: S2O3D corresponds to S_2_O_3_D; SO3R corresponds to hSO_3_; _pilexp_ refers to replicate 4.
**Figure S3:** Overlapping protein groups identified across three biological replicates (R1–R3) for thiosulfate disproportionation ‐S_2_O_3_D‐ (A, B) and four biological replicates (R1–R4) for cultures carried out in hydrogenotrophic sulfite‐containing media ‐hSO_3_‐ (C, D). The left diagram shows the membrane fraction (Memb), while the right diagram represents the soluble fraction (Sol).
**Figure S4:** Genomic organisation of the key genes discussed in this study.
**Figure S5:** Venn diagrams illustrating proteins identified in strain S69ᵀ in soluble ‐Sol‐ and membrane ‐Memb‐ fractions under S_2_O_3_D (A) and in the hSO_3_ condition (B).
**Figure S6:** Heatmap showing the relative abundance of proteins (fold change ≥ 1.5; adjusted *p*‐value ≤ 0.05). A detailed list of these proteins is provided in Supplementary Table S8.

**Table S1:** Main report from DIA‐NN software.
**Table S2:** Peptides identified per protein using DIA‐NN software.
**Table S3:** Protein group abundance matrix per sample generated by DIA‐NN software.
**Table S4:** Protein abundance in each total fraction (Total = Sol + Memb) and statistical confidence obtained using the MCQR package.
**Table S5:** Hypothesis test results obtained with the MCQR package (adjusted *p*‐value ≤ 0.05).
**Table S6:** Significantly differentially abundant proteins under thiosulfate disproportionation (S_2_O_3_D) and in the hydrogenotrophic sulfite‐containing (hSO_3_) condition.
**Table S7:** List of heatmap proteins in descending order (fold change ≥ 1.5 and an adjusted *p*‐value ≤ 0.05; 717 proteins).
**Table S8:** Unique proteins identified during thiosulfate disproportionation (S_2_O_3_D) and in the hydrogenotrophic sulfite‐containing (hSO_3_) condition.
**Table S9:** Genetic characteristics of differentially abundant molybdoenzymes under S_2_O_3_D or hSO_3_ conditions and comparison to known enzymes. Legend: aa, amino acids; TM, transmembrane helice; C‐ter: C‐terminus; tat, twin‐arginine translocase leader (tat leader) sequence located at the N‐terminus of the catalytic subunit of molybdoenzymes, involved in targeting these enzymes to the periplasm; SD, strain with known ability for sulfur compound disproportionation^#^.?SD, a strain for which the ability to disproportionate sulfur compounds has never been investigated^#^. NSD, strain with known unability for sulfur compound disproportionation^#^.
**Table S10:** Genetic characteristics of hydroxylamine reductase and Hdr‐like/mvh complex.

## Data Availability

The mass spectrometry proteomic data are available in PRIDE repository under the dataset identifier [PXD069670].

## References

[emi470406-bib-0001] Alain, K. , H. S. Aronson , M. Allioux , S. Yvenou , and J. P. Amend . 2022. “Sulfur Disproportionation Is Exergonic in the Vicinity of Marine Hydrothermal Vents.” Environmental Microbiology 24: 2210–2219. 35315563 10.1111/1462-2920.15975

[emi470406-bib-0002] Allioux, M. , S. Yvenou , A. Godfroy , Z. Shao , M. Jebbar , and K. Alain . 2022. “Genome Analysis of a New Sulphur Disproportionating Species *Thermosulfurimonas Strain* F29 and Comparative Genomics of Sulfur‐Disproportionating Bacteria From Marine Hydrothermal Vents.” Microbial Genomics 8: mgen.0.000865. 10.1099/mgen.0.000865 PMC9676032 36136081

[emi470406-bib-0003] Allioux, M. , S. Yvenou , G. Slobodkina , et al. 2020. “Genomic Characterization and Environmental Distribution of a Thermophilic Anaerobe *Dissulfurirhabdus Thermomarina* SH388T Involved in Disproportionation of Sulfur Compounds in Shallow Sea Hydrothermal Vents.” Microorganisms 8: 1132. 32727039 10.3390/microorganisms8081132 PMC7463578

[emi470406-bib-0004] Appel, L. , M. Willistein , C. Dahl , U. Ermler , and M. Boll . 2021. “Functional Diversity of Prokaryotic HdrA(BC) Modules: Role in Flavin‐Based Electron Bifurcation Processes and Beyond.” Biochimica et Biophysica Acta, Bioenergetics 1862: 148379. 33460586 10.1016/j.bbabio.2021.148379

[emi470406-bib-0005] Aronson, H. S. , C. E. Clark , D. E. LaRowe , J. P. Amend , L. Polerecky , and J. L. Macalady . 2023. “Sulfur Disproportionating Microbial Communities in a Dynamic, Microoxic‐Sulfidic Karst System.” Geobiology 21: 791–803. 37721188 10.1111/gbi.12574

[emi470406-bib-0006] Balliau, T. , A. Frambourg , O. Langella , M.‐L. Martin , M. Zivy , and M. Blein‐Nicolas . 2025. “MCQR: Enhancing the Processing and Analysis of Quantitative Proteomics Data by Incorporating Chromatography and Mass Spectrometry Information.” Journal of Proteome Research 24: 2861–2873. 40391828 10.1021/acs.jproteome.4c01119

[emi470406-bib-0007] Blum, M. , A. Andreeva , L. C. Florentino , et al. 2025. “InterPro: The Protein Sequence Classification Resource in 2025.” Nucleic Acids Research 53: D444–D456. 39565202 10.1093/nar/gkae1082 PMC11701551

[emi470406-bib-0008] Bradford, M. M. 1976. “A Rapid and Sensitive Method for the Quantification of Microgram Quantities of Protein Utilizing the Principle of Protein‐Dye Binding.” Analytical Biochemistry 72: 248–254. 942051 10.1016/0003-2697(76)90527-3

[emi470406-bib-0010] Cline, J. D. 1969. “Spectrophotometric Determination of Hydrogen Sulfide in Natural Waters.” Limnology and Oceanography 14: 454–458.

[emi470406-bib-0011] Cuevasanta, E. , A. Denicola , B. Alvarez , and M. N. Möller . 2012. “Solubility and Permeation of Hydrogen Sulfide in Lipid Membranes.” PLoS One 7: e34562. 22509322 10.1371/journal.pone.0034562 PMC3324494

[emi470406-bib-0012] Dahl, C. 2020. “A Biochemical View on the Biological Sulfur Cycle.” In Environmental Technologies to Treat Sulfur Pollution: Principles and Engineering, edited by P. N. L. Lens , 55–96. IWA Publishing ISBN 9781789060966.

[emi470406-bib-0013] Darzi, Y. , I. Letunic , P. Bork , and T. Yamada . 2018. “iPath3.0: Interactive Pathways Explorer v3.” Nucleic Acids Research 46: W510–W513. 29718427 10.1093/nar/gky299 PMC6031023

[emi470406-bib-0014] Degré, G. , A. Tempier , M. Vaillant , et al. 2026. “Demonstration of the Role of Both a Ttr and a Psr Homologue Enzymes in the Respiration of Tetrathionate by an Environmental Bacterium *Shewanella* sp. ANA‐3.” Environmental Microbiology 28: e70258. 41761654 10.1111/1462-2920.70258 PMC12949373

[emi470406-bib-0015] Demichev, V. , C. B. Messner , S. I. Vernardis , K. S. Lilley , and M. Ralser . 2020. “DIA‐NN: Neural Networks and Interference Correction Enable Deep Proteome Coverage in High Throughput.” Nature Methods 17: 41–44. 31768060 10.1038/s41592-019-0638-x PMC6949130

[emi470406-bib-0016] D'Hondt, S. , F. Inagaki , B. Orcutt , and K.‐U. Hinrichs . 2019. “IODP Advances in the Understanding of Subseafloor Life.” Oceanography 32: 198–207.

[emi470406-bib-0017] Dick, G. J. 2019. “The Microbiomes of Deep‐Sea Hydrothermal Vents: Distributed Globally, Shaped Locally.” Nature Reviews. Microbiology 17: 271–283. 30867583 10.1038/s41579-019-0160-2

[emi470406-bib-0018] Digel, L. , G. Ceriotti , L. M. Keller , et al. 2026. “Breathing Both Ways: Simultaneous Aerobic–Anaerobic Respiration in Microbes.” Trends in Microbiology 34: 851–863. 41966861 10.1016/j.tim.2026.03.010

[emi470406-bib-0019] Dörries, M. , L. Wöhlbrand , M. Kube , R. Reinhardt , and R. Rabus . 2016. “Genome and Catabolic Subproteomes of the Marine, Nutritionally Versatile, Sulfate‐Reducing Bacterium *Desulfococcus multivorans* DSM 2059.” BMC Genomics 17: 918. 27846794 10.1186/s12864-016-3236-7 PMC5109826

[emi470406-bib-0020] Duarte, A. G. , A. C. C. Barbosa , D. Ferreira , G. Manteigas , R. M. Domingos , and I. A. C. Pereira . 2021. “Redox Loops in Anaerobic Respiration ‐ the Role of the Widespread NrfD Protein Family and Associated Dimeric Redox Module.” Biochimica et Biophysica Acta ‐ Bioenergetics 1862: 148416. 33753023 10.1016/j.bbabio.2021.148416

[emi470406-bib-0021] Eddy, S. R. 2011. “Accelerated Profile HMM Searches.” PLoS Computational Biology 7: 1–16. 10.1371/journal.pcbi.1002195 PMC3197634 22039361

[emi470406-bib-0022] Ferreira, D. , A. C. C. Barbosa , G. P. Oliveira , T. Catarino , S. S. Venceslau , and I. A. C. Pereira . 2022. “The DsrD Functional Marker Protein Is an Allosteric Activator of the DsrAB Dissimilatory Sulfite Reductase.” Proceedings of the National Academy of Sciences of The United States of America 119: e2118880119. 35064091 10.1073/pnas.2118880119 PMC8794893

[emi470406-bib-0023] Finster, K. 2008. “Microbiological Disproportionation of Inorganic Sulfur Compounds.” Journal of Sulfur Chemistry 29: 281–292.

[emi470406-bib-0024] Florentino, A. P. , I. A. C. Pereira , S. Boeren , M. Van Den Born , A. J. M. Stams , and I. Sánchez‐Andrea . 2019. “Insight Into the Sulfur Metabolism of *Desulfurella Amilsii* by Differential Proteomics.” Environmental Microbiology 21: 209–225. 30307104 10.1111/1462-2920.14442 PMC6378623

[emi470406-bib-0025] Fossing, H. , and B. B. Jørgensen . 1990. “Oxidation and Reduction of Radiolabeled Inorganic Sulfur Compounds in an Estuarine Sediment, Kysing Fjord, Denmark.” Geochimica et Cosmochimica Acta 54: 2731–2742.

[emi470406-bib-0026] Frolova, A. A. , G. B. Slobodkina , R. V. Baslerov , A. A. Novikov , E. A. Bonch‐Osmolovskaya , and A. I. Slobodkin . 2018. “ *Thermosulfurimonas Marina* sp. Nov., an Autotrophic Sulfur‐Disproportionating and Nitrate‐Reducing Bacterium Isolated From a Shallow‐Sea Hydrothermal Vent.” Microbiology 87: 502–507.

[emi470406-bib-0086] Haja, D. K. , C. H. Wu , O. Ponomarenko , F. L. Poole, II , G. N. George , and M. W. W. Adams . 2020. “Improving Arsenic Tolerance of Pyrococcus Furiosus by Heterologous Expression of a Respiratory Arsenate Reductase.” Applied and Environmental Microbiology 86: e01728‐20. 32859593 10.1128/AEM.01728-20 PMC7580539

[emi470406-bib-0027] Hallgren, J. , K. D. Tsirigos , M. D. Pedersen , et al. 2022. “DeepTMHMM Predicts Alpha and Beta Transmembrane Proteins Using Deep Neural Networks.” bioRxiv: The Preprint Server for Biology.

[emi470406-bib-0028] Hansen, A. H. , L. G. Lorentzen , D. J. Leeming , J. M. B. Sand , P. Hägglund , and M. J. Davies . 2025. “Peptidomic and Proteomic Analysis of Precision‐Cut Lung Slice Supernatants.” Analytical Biochemistry 702: 115837. 40058539 10.1016/j.ab.2025.115837

[emi470406-bib-0029] Hashimoto, Y. , S. Shimamura , A. Tame , et al. 2022. “Physiological and Comparative Proteomic Characterization of *Desulfolithobacter Dissulfuricans* Gen. Nov., sp. Nov., a Novel Mesophilic, Sulfur‐Disproportionating Chemolithoautotroph From a Deep‐Sea Hydrothermal Vent.” Frontiers in Microbiology 13: 1042116. 36532468 10.3389/fmicb.2022.1042116 PMC9751629

[emi470406-bib-0030] Hemon, M. , L. Novák , M. Allioux , L. Ailliot , E. Vince , and K. Alain . 2025. “Draft Genome Sequence of *Desulfobacterota* Strain M19, a Mesophilic Sulfur‐Disproportionating Bacterium From a Deep‐Sea Hydrothermal Vent on the Mid‐Atlantic Ridge.” Microbiology Resource Announcements 14: e00295‐25. 40488495 10.1128/mra.00295-25 PMC12243485

[emi470406-bib-0031] Hensel, M. , A. P. Hinsley , T. Nikolaus , G. Sawers , and B. C. Berks . 1999. “The Genetic Basis of Tetrathionate Respiration in *Salmonella typhimurium* .” Molecular Microbiology 32: 275–287. 10231485 10.1046/j.1365-2958.1999.01345.x

[emi470406-bib-0032] Hille, R. , J. Hall , and P. Basu . 2014. “The Mononuclear Molybdenum Enzymes.” Chemical Reviews 114: 3963–4038. 24467397 10.1021/cr400443z PMC4080432

[emi470406-bib-0033] Hughes, C. S. , S. Moggridge , T. Müller , P. H. Sorensen , G. B. Morin , and J. Krijgsveld . 2019. “Single‐Pot, Solid‐Phase‐Enhanced Sample Preparation for Proteomics Experiments.” Nature Protocols 14: 68–85. 30464214 10.1038/s41596-018-0082-x

[emi470406-bib-0034] Johnston, D. T. , B. A. Wing , J. Farquhar , et al. 2005. “Active Microbial Sulfur Disproportionation in the Mesoproterozoic.” Science 310: 1477–1479. 16322453 10.1126/science.1117824

[emi470406-bib-0035] Jørgensen, B. B. 1990. “A Thiosulfate Shunt in the Sulfur Cycle of Marine Sediments.” Science 249: 152–154. 17836966 10.1126/science.249.4965.152

[emi470406-bib-0036] Keller, L. M. , D. R. Colman , and E. S. Boyd . 2025. “Simultaneous Aerobic and Anaerobic Respiration in Hot Spring Chemolithotrophic Bacteria.” Nature Communications 16: 1063. 10.1038/s41467-025-56418-4 PMC11772811 39870657

[emi470406-bib-0037] Kelley, D. S. , J. A. Baross , and J. R. Delaney . 2002. “Volcanoes, Fluids, and Life at Mid‐Ocean Ridge Spreading Centers.” Annual Review of Earth and Planetary Sciences 30: 385–491.

[emi470406-bib-0038] Kimura, H. 2015. “Hydrogen Sulfide and Polysulfides as Signaling Molecules.” Proceedings of the Japan Academy. Series B, Physical and Biological Sciences 91: 131–159. 25864468 10.2183/pjab.91.131 PMC4568289

[emi470406-bib-0039] Krämer, M. , and H. Cypionka . 1989. “Sulfate Formation via ATP Sulfurylase in Thiosulfate‐ and Sulfite‐Disproportionating Bacteria.” Archives of Microbiology 151: 232–237.

[emi470406-bib-0040] Kurth, J. M. , C. Dahl , and J. N. Butt . 2015. “Catalytic Protein Film Electrochemistry Provides a Direct Measure of the Tetrathionate/Thiosulfate Reduction Potential.” Journal of the American Chemical Society 137: 13232–13235. 26437022 10.1021/jacs.5b08291

[emi470406-bib-0041] Lustermans, J. J. M. , N. Basu , L. Digel , and K. Aiyer . 2025. “Iron Reduction Under Oxic Conditions by *Microbacterium Deferre* sp. Nov. A1‐JK(T).” Nature Communications 16: 6183. 10.1038/s41467-025-61424-7 PMC12227724 40615380

[emi470406-bib-0042] Lyutvinskiy, Y. , H. Yang , D. Rutishauser , and R. A. Zubarev . 2013. “In Silico Instrumental Response Correction Improves Precision of Label‐Free Proteomics and Accuracy of Proteomics‐Based Predictive Models.” Molecular & Cellular Proteomics 12: 2324–2331. 23589346 10.1074/mcp.O112.023804 PMC3734588

[emi470406-bib-0043] Mardanov, A. V. , A. V. Beletsky , V. V. Kadnikov , A. I. Slobodkin , and N. V. Ravin . 2016. “Genome Analysis of *Thermosulfurimonas Dismutans*, the First Thermophilic Sulfur‐Disproportionating Bacterium of the Phylum *Thermodesulfobacteria* .” Frontiers in Microbiology 7: 950. 27379079 10.3389/fmicb.2016.00950 PMC4911364

[emi470406-bib-0044] Mathai, J. C. , A. Missner , P. Kügler , et al. 2009. “No Facilitator Required for Membrane Transport of Hydrogen Sulfide.” Proceedings of the National Academy of Sciences of The United States of America 106: 16633–16638. 19805349 10.1073/pnas.0902952106 PMC2757810

[emi470406-bib-0045] Muramatsu, F. , M. Tonomura , M. Yamada , et al. 2020. “Possible Involvement of a Tetrathionate Reductase Homolog in Dissimilatory Arsenate Reduction by *Anaeromyxobacter* sp. Strain PSR‐1.” Applied and Environmental Microbiology 86: e00829‐20. 32978134 10.1128/AEM.00829-20 PMC7657633

[emi470406-bib-0046] Neukirchen, S. , I. A. C. Pereira , and F. L. Sousa . 2023. “Stepwise Pathway for Early Evolutionary Assembly of Dissimilatory Sulfite and Sulfate Reduction.” ISME Journal 17: 1680–1692. 37468676 10.1038/s41396-023-01477-y PMC10504309

[emi470406-bib-0047] Neukirchen, S. , and F. L. Sousa . 2021. “DiSCo: A Sequence‐Based Type‐Specific Predictor of Dsr‐Dependent Dissimilatory Sulphur Metabolism in Microbial Data.” Microbial Genomics 7: 000603. 34241589 10.1099/mgen.0.000603 PMC8477390

[emi470406-bib-0048] Novak, L. V. F. , L. Jiang , M. Hemon , et al. 2026. “Sulfur Disproportionation Occurs Globally Across Anoxic Habitats and Has Multiple Mechanisms of Independent Evolutionary Origin.” ISME Journal 20: wrag042. 41766620 10.1093/ismejo/wrag042 PMC12998232

[emi470406-bib-0049] Padalko, A. , V. Karavaeva , J. Zamarreno Beas , S. Neukirchen , and F. L. Sousa . 2025. “Bioenergetics Evolution: The Link Between Earth's and Life's History.” Philosophical Transactions of the Royal Society of London. Series B, Biological Sciences 380: 20240102. 40770987 10.1098/rstb.2024.0102 PMC12329440

[emi470406-bib-0050] Parey, K. , U. Demmer , E. Warkentin , A. Wynen , U. Ermler , and C. Dahl . 2013. “Structural, Biochemical and Genetic Characterization of Dissimilatory ATP Sulfurylase From *Allochromatium vinosum* .” PLoS One 8: e74707. 24073218 10.1371/journal.pone.0074707 PMC3779200

[emi470406-bib-0051] Paysan‐Lafosse, T. , A. Andreeva , M. Blum , et al. 2025. “The Pfam Protein Families Database: Embracing AI/ML.” Nucleic Acids Research 53: D523–D534. 39540428 10.1093/nar/gkae997 PMC11701544

[emi470406-bib-0052] Petushkova, E. , S. Iuzhakov , and A. Tsygankov . 2019. “Differences in Possible TCA Cycle Replenishing Pathways in Purple Non‐Sulfur Bacteria Possessing Glyoxylate Pathway.” Photosynthesis Research 139: 523–537. 30219941 10.1007/s11120-018-0581-1

[emi470406-bib-0053] Philippot, P. , M. Van Zuilen , K. Lepot , C. Thomazo , J. Farquhar , and M. J. Van Kranendonk . 2007. “Early Archaean Microorganisms Preferred Elemental Sulfur, Not Sulfate.” Science 317: 1534–1537. 17872441 10.1126/science.1145861

[emi470406-bib-0054] Piłsyk, S. , and A. Paszewski . 2009. “Sulfate Permeases Phylogenetic Diversity of Sulfate Transport.” Acta Biochimica Polonica 56: 375–384. 19724780

[emi470406-bib-0055] Plum‐Jensen, L. E. , M. G. Mohr , T. S. Tanabe , et al. 2026. “Distribution of a Novel DsrEFH Sulfur Transferase Suggests Widespread Sulfur Oxidation Capacity in Sulfate Reducers.” ISME Journal 23: wrag130. 10.1093/ismejo/wrag130 PMC13293266 42176189

[emi470406-bib-0056] Ramos, A. R. , K. L. Keller , J. D. Wall , and I. A. C. Pereira . 2012. “The Membrane QmoABC Complex Interacts Directly With the Dissimilatory Adenosine 5′‐Phosphosulfate Reductase in Sulfate Reducing Bacteria.” Frontiers in Microbiology 3: 137. 22536198 10.3389/fmicb.2012.00137 PMC3333476

[emi470406-bib-0057] Riahi, S. , and C. N. Rowley . 2014. “Why Can Hydrogen Sulfide Permeate Cell Membranes?” Journal of the American Chemical Society 136: 15111–15113. 25323018 10.1021/ja508063s

[emi470406-bib-0058] Rothery, R. A. , G. J. Workun , and J. H. Weiner . 2008. “The Prokaryotic Complex Iron‐Sulfur Molybdoenzyme Family.” Biochimica et Biophysica Acta 1778: 1897–1929. 17964535 10.1016/j.bbamem.2007.09.002

[emi470406-bib-0059] Santos, A. A. , S. S. Venceslau , F. Grein , et al. 2015. “A Protein Trisulfide Couples Dissimilatory Sulfate Reduction to Energy Conservation.” Science 350: 1541–1545. 26680199 10.1126/science.aad3558

[emi470406-bib-0060] Shirodkar, S. , S. Reed , M. Romine , and D. Saffarini . 2011. “The Octahaem SirA Catalyses Dissimilatory Sulfite Reduction in *Shewanella oneidensis* MR‐1.” Environmental Microbiology 13: 108–115. 21199252 10.1111/j.1462-2920.2010.02313.x

[emi470406-bib-0061] Simon, J. , and P. M. H. Kroneck . 2013. “Microbial Sulfite Respiration.” Advances in Microbial Physiology 62: 45–117. 23481335 10.1016/B978-0-12-410515-7.00002-0

[emi470406-bib-0062] Slobodkin, A. I. , A.‐L. Reysenbach , G. B. Slobodkina , et al. 2012. “ *Thermosulfurimonas Dismutans* Gen. Nov., sp. Nov., an Extremely Thermophilic Sulfur‐Disproportionating Bacterium From a Deep‐Sea Hydrothermal Vent.” International Journal of Systematic and Evolutionary Microbiology 62: 2565–2571. 22199218 10.1099/ijs.0.034397-0

[emi470406-bib-0063] Slobodkin, A. I. , A.‐L. Reysenbach , G. B. Slobodkina , T. V. Kolganova , N. A. Kostrikina , and E. A. Bonch‐Osmolovskaya . 2013. “ *Dissulfuribacter Thermophilus* Gen. Nov., sp. Nov., a Thermophilic, Autotrophic, Sulfur‐Disproportionating, Deeply Branching Deltaproteobacterium From a Deep‐Sea Hydrothermal Vent.” International Journal of Systematic and Evolutionary Microbiology 63: 1967–1971. 23024145 10.1099/ijs.0.046938-0

[emi470406-bib-0064] Slobodkin, A. I. , and G. B. Slobodkina . 2019. “Diversity of Sulfur‐Disproportionating Microorganisms.” Microbiology 88: 509–522.

[emi470406-bib-0065] Slobodkin, A. I. , G. B. Slobodkina , A. N. Panteleeva , N. A. Chernyh , A. A. Novikov , and E. A. Bonch‐Osmolovskaya . 2016. “ *Dissulfurimicrobium Hydrothermale* Gen. Nov., sp. Nov., a Thermophilic, Autotrophic, Sulfur‐Disproportionating Deltaproteobacterium Isolated From a Hydrothermal Pond.” International Journal of Systematic and Evolutionary Microbiology 66: 1022–1026. 26646853 10.1099/ijsem.0.000828

[emi470406-bib-0066] Slobodkina, G. B. , A.‐L. Reysenbach , T. V. Kolganova , A. A. Novikov , E. A. Bonch‐Osmolovskaya , and A. I. Slobodkin . 2017. “ *Thermosulfuriphilus Ammonigenes* Gen. Nov., sp. Nov., a Thermophilic, Chemolithoautotrophic Bacterium Capable of Respiratory Ammonification of Nitrate With Elemental Sulfur.” International Journal of Systematic and Evolutionary Microbiology 67: 3474–3479. 28857038 10.1099/ijsem.0.002142

[emi470406-bib-0067] Sorokin, D. Y. , A. Y. Merkel , R. H. Ziganshin , and I. V. Kublanov . 2025. “Growth Physiology, Genomics, and Proteomics of *Desulfurivibrio Dismutans* sp. Nov., an Obligately Chemolithoautotrophic, Sulfur Disproportionating and Ammonifying Haloalkaliphile From Soda Lakes.” Frontiers in Microbiology 16: 1590477. 40485835 10.3389/fmicb.2025.1590477 PMC12142623

[emi470406-bib-0068] Stoffels, L. , M. Krehenbrink , B. C. Berks , and G. Unden . 2012. “Thiosulfate Reduction in *Salmonella enterica* Is Driven by the Proton Motive Force.” Journal of Bacteriology 194: 475–485. 22081391 10.1128/JB.06014-11 PMC3256639

[emi470406-bib-0069] Szklarczyk, D. , R. Kirsch , M. Koutrouli , et al. 2023. “The STRING Database in 2023: Protein–Protein Association Networks and Functional Enrichment Analyses for Any Sequenced Genome of Interest.” Nucleic Acids Research 51: D638–D646. 36370105 10.1093/nar/gkac1000 PMC9825434

[emi470406-bib-0071] Teufel, F. , J. J. Almagro Armenteros , A. R. Johansen , et al. 2022. “SignalP 6.0 Predicts All Five Types of Signal Peptides Using Protein Language Models.” Nature Biotechnology 40: 1023–1025. 10.1038/s41587-021-01156-3 PMC9287161 34980915

[emi470406-bib-0072] Thauer, R. K. , K. Jungermann , and K. Decker . 1977. “Energy Conservation in Chemotrophic Anaerobic Bacteria.” Bacteriological Reviews 41: 100–180. 860983 10.1128/br.41.1.100-180.1977 PMC413997

[emi470406-bib-0073] The UniProt Consortium . 2025. “UniProt: The Universal Protein Knowledgebase in 2025.” Nucleic Acids Research 53: D609–D617. 39552041 10.1093/nar/gkae1010 PMC11701636

[emi470406-bib-0074] Vallenet, D. , A. Calteau , M. Dubois , et al. 2020. “MicroScope: An Integrated Platform for the Annotation and Exploration of Microbial Gene Functions Through Genomic, Pangenomic and Metabolic Comparative Analysis.” Nucleic Acids Research 48: D579–D589. 31647104 10.1093/nar/gkz926 PMC7145621

[emi470406-bib-0075] Valot, B. , O. Langella , E. Nano , and M. Zivy . 2011. “MassChroQ: A Versatile Tool for Mass Spectrometry Quantification.” Proteomics 11: 3572–3577. 21751374 10.1002/pmic.201100120

[emi470406-bib-0076] Vink, B. W. 1996. “Stability Relations of Antimony and Arsenic Compounds in the Light of Revised and Extended Eh‐pH Diagrams.” Chemical Geology 130: 21–30.

[emi470406-bib-0077] Wacey, D. , M. R. Kilburn , M. Saunders , J. Cliff , and M. D. Brasier . 2011. “Microfossils of Sulphur‐Metabolizing Cells in 3.4‐Billion‐Year‐Old Rocks of Western Australia.” Nature Geoscience 4: 698–702.

[emi470406-bib-0078] Wang, S. , L. Jiang , S. Xie , et al. 2023. “Disproportionation of Inorganic Sulfur Compounds by Mesophilic Chemolithoautotrophic *Campylobacterota* .” mSystems 8: e00954‐22. 36541763 10.1128/msystems.00954-22 PMC9948710

[emi470406-bib-0079] Wasmund, K. , M. Mußmann , and A. Loy . 2017. “The Life Sulfuric: Microbial Ecology of Sulfur Cycling in Marine Sediments.” Environmental Microbiology Reports 9: 323–344. 28419734 10.1111/1758-2229.12538 PMC5573963

[emi470406-bib-0080] Wu, X.‐T. , M. Qiu , Y.‐Q. He , et al. 2025. “Disproportionation of Elemental Sulfur by Exiguobacterium From Marine Sediment.” ISME Communications 5: ycaf168. 41080528 10.1093/ismeco/ycaf168 PMC12510457

[emi470406-bib-0081] Xu, G. , H. Yang , J. Han , et al. 2024. “Regulatory Roles of Extracellular Polymeric Substances in Uranium Reduction via Extracellular Electron Transfer by *Desulfovibrio vulgaris* UR1.” Environmental Research 262: 119862. 39208974 10.1016/j.envres.2024.119862

[emi470406-bib-0082] Yu, N. Y. , J. R. Wagner , M. R. Laird , et al. 2010. “PSORTb 3.0: Improved Protein Subcellular Localization Prediction With Refined Localization Subcategories and Predictive Capabilities for All Prokaryotes.” Bioinformatics 26: 1608–1615. 20472543 10.1093/bioinformatics/btq249 PMC2887053

[emi470406-bib-0083] Yvenou, S. , M. Allioux , A. Slobodkin , G. Slobodkina , M. Jebbar , and K. Alain . 2022. “Genetic Potential of *Dissulfurimicrobium Hydrothermale*, an Obligate Sulfur‐Disproportionating Thermophilic Microorganism.” Microorganisms 10: 60. 10.3390/microorganisms10010060 PMC8780430 35056509

[emi470406-bib-0084] Zeng, X. , K. Alain , and Z. Shao . 2021. “Microorganisms From Deep‐Sea Hydrothermal Vents.” Marine Life Science & Technology 3: 204–230. 37073341 10.1007/s42995-020-00086-4 PMC10077256

[emi470406-bib-0085] Zhou, Z. , P. Q. Tran , E. S. Cowley , E. Trembath‐Reichert , and K. Anantharaman . 2025. “Diversity and Ecology of Microbial Sulfur Metabolism.” Nature Reviews Microbiology 23: 122–140. 39420098 10.1038/s41579-024-01104-3

