# Supplementary material for: Advancing the Resolution of the Sulfur Compound Disproportionation Puzzle in Dissulfuribacter Thermophilus S69ᵀ Through Quantitative Label‐Free Comparative Proteomics
Source: Environ Microbiol Rep. 2026 Aug 19;18(4):e70406. doi: 10.1111/1758-2229.70406 (PMC13490349; doi:10.1111/1758-2229.70406)
Supplement: Supplementary file 1 [file EMI4-18-e70406-s001.docx]

**Supplementary Information**

**Advancing the resolution of the sulfur compound disproportionation puzzle in *Dissulfuribacter thermophilus* S69ᵀ through quantitative label-free comparative proteomics**

**Running title:** Sulfur metabolism of *Dissulfuribacter thermophilus*

Marilina Fernandez^1*^ | Barbara Schoepp-Cothenet^2^ | Marie Hemon^3^ | Carine Rodrigues-Machado^1^ | Lukas V.F. Novák^3^ | Xavier Philippon^3^ | Sébastien Laurent^3^ | Céline Henry^1^ | Karine Alain^3*^

^1^Université Paris-Saclay, INRAE, AgroParisTech, Micalis Institute, PAPPSO Platform, 78350, Jouy-en-Josas, France |

^2^Aix-Marseille Université, CNRS, BIP-UMR 7281, IMM, IM2B, Marseille, France | ^3^Univ Brest, CNRS, Ifremer, EMR 6002 BIOMEX, IRP 1211 MicrobSea, Unité Biologie et Ecologie des Ecosystèmes marins Profonds BEEP, F-29280 Plouzané, France.

* Correspondence: Marilina Fernandez (mfernandez@exa.unrc.edu.ar) & Karine Alain (Karine.Alain@univ-brest.fr)

ORCID: FM, 0000-0002-7471-6189; BSC, [0000-0002-6820-4478](https://orcid.org/0000-0002-6820-4478) ; HM, 0009-0001-4765-6483; RMC, 0000-0003-3675-6267; NLVF, 0000-0001-7773-9119; PX, 0009-0004-3555-633X; HC, [0000-0002-2355-1791](http://orcid.org/0000-0002-2355-1791); KA, 0000-0002-8812-9081

**Materials and Methods**

**Cell counts**

Bacterial growth was assessed by direct cell counting under an Olympus BX60 epifluorescence microscope after fixation and staining for 5 min. of 300 µL culture in a reaction mixture containing 75 µL of 25 % glutaraldehyde and 300 µL of 0.1% acridine orange (Sigma-Aldrich), and then filtration onto 0.2 μm polycarbonate membranes (Isopore, black, Millipore). The prepared filters were placed between slide and coverslip and observed with a 492-577 nm green WG filter under UV, objective ×100. Cell counts were conducted in a square with an area of 0.0025 µm^2^. Within this known-area grid, cells were counted for at least 20 randomly chosen squares, and by counting a minimum number of 300 cells.

**Anion analysis by ion chromatography**

A Thermo Scientific™ Dionex™ ICS-6000 HPIC™ system was used, equipped with a microbore flow controller (1–3 mm column internal diameter), an ion exchange column (Dionex™ IonPac™ AG15, 2 mm × 50 mm), and a suppressor (Thermo Scientific™ Dionex™ ADRS™ 600). Potassium hydroxide (KOH) was employed as the eluent at a flow rate of 0.3 mL·min⁻¹. The gradient program was as follows: 8 mM KOH for 3 min; increased to 14 mM over 12 min; to 17 mM over 8 min; to 45 mM over 0.5 min; to 48.2 mM over 10.5 min; to 58 mM over 6 min; to 80 mM over 9.75 min; and finally decreased back to 8 mM over 10 min.

**Biomass collection, cell fractionation and protein extraction**

Cells grown under S_2_O_3_D and under hSO₃ were harvested at mid-exponential phase (42 h and 12 h, respectively). Cultures were centrifuged at 7440 × g for 20 min at 4 °C, and cell pellets were washed with a rinsing solution (25 g/L NaCl, 4.4 g/L MgCl_2_). For thiosulfate-grown samples, a preliminary low-speed centrifugation step (200 g, 5 min, 4 °C) was performed to remove residual iron from the preculture. Washed cells were stored at –80 °C until fractionation.

For cell fractionation, pellets were resuspended in lysis buffer (50 mM Tris-HCl, pH 8.0) supplemented with phosphatase and protease inhibitor cocktails (Thermo Scientific and Roche, respectively), added according to the manufacturers’ instructions. Cell disruption was performed at 2.5 Kbar using a high-pressure cell disruptor (Constant Systems, UK). The lysate was centrifuged at 7440 × g for 15 min at 4 °C to remove cell debris, and the resulting supernatant was subjected to ultracentrifugation at 257 000 × g for 50 min at 4 °C.

The supernatant was retained as the soluble protein fraction. The pellet—enriched in membrane and residual soluble proteins—was resuspended in 300 μL of DUTT buffer adjusted to pH 8.0 (5 mM dithiothreitol (DTT), 6 M urea, 2 M thiourea, 100 mM Tris-HCl, 10 μL/mL phosphatase and 1pill/10mL protease inhibitors). To enhance solubilization, this membrane fraction was sonicated for 1 min, then centrifuged at 7440 × g for 2 min at 4 °C. Protein concentrations in the supernatant were determined using the Bradford assay (Bradford, 1976), using bovine serum albumin (BSA) as the standard.

**Shotgun proteomics sample preparation**

Sample preparation for nanoLC-MS/MS followed the SP3 protocol (Hughes et al., 2019; Hansen et a, 2025). Briefly, 5 µg of proteins were reduced with 10 mM DTT at 56 °C for 30 min, followed by alkylation with 200 mM iodoacetamide for 30 min at room temperature in the dark. The reaction was quenched with 10 mM DTT. Proteins were bound to carboxylate-coated magnetic beads (Sera-Mag™, Cytiva, Sweden) at a 16:1 bead:protein ratio. Binding was facilitated by the addition of formic acid, followed by acetonitrile (ACN) to a final concentration of 50%. Beads were washed twice with 70% ethanol. On-bead digestion was performed using trypsin (Promega, Madison, USA) at a 1:50 enzyme:protein ratio in 10 µL of 50 mM ammonium bicarbonate (pH 8), and incubated overnight at 37 °C. Peptides were sequentially eluted with 95% ACN and then 2% ACN in water, dried using a SpeedVac concentrator, and stored at −20 °C. Prior to mass spectrometry analysis, the dried peptides were reconstituted in a loading buffer containing 98% Hl. 2O, 2% ACN, and 0.1% formic acid (FA) and the quantity injected was adjusted to achieve optimal signal intensity (1-1.5E6).

**Results**

**Identification of the Wood–Ljungdahl pathway (WLP)**

The WLP pathway includes a formate dehydrogenase (DITHS69_2496; DITHS69_2497; DITHS69_1351; DITHS69_2206), a formate tetrahydrofolate ligase (DITHS69_0017), a bifunctional methylenetetrahydrofolate dehydrogenase/cyclohydrolase (DITHS69_0015), a 5-methyltetrahydrofolate corrinoid/iron-sulfur protein methyltransferase (DITHS69_0003), a carbon-monoxide dehydrogenase (DITHS69_0014; DITHS69_1462), a 5,10-methylenetetrahydrofolate reductase (DITHS69_0006; DITHS69_0049; DITHS69_0595; DITHS69_1318; DITHS69_1653) and a CO-methylating acetyl-CoA synthase (DITHS69_0005).

Bradford, M. M. (1976). A rapid and sensitive method for the quantitation of microgram quantities of protein utilizing the principle of protein-dye binding. *Anal. Biochem*., 72, 248–254. <https://doi.org/10.1016/0003-2697(76)90527-3>

Hansen, A. H., et al. (2025). Peptidomic and proteomic analysis of precision-cut lung slice supernatants. *Anal. Biochem*., 702, 115837. <https://doi.org/10.1016/j.ab.2025.115837>

Hughes, C. S., et al. (2019). Single-pot, solid-phase-enhanced sample preparation for proteomics experiments. *Nat. Protoc*., 14(1), 68–85. https://doi.org/10.1038/s41596-018-0089-5

**FIGURE S1 |** Growth of strain S69^T^ via disproportionation of thiosulfate (A), and under hydrogenotrophic sulfite-containing (hSO3) conditions (B). Full growth curves covering all growth phases are shown in grey, while the growth kinetics for proteomic sampling are shown in black. Proteins were extracted from the last black point on each curve, corresponding to the mid-exponential growth phase. Temporal changes in thiosulfate, sulfate, sulfide, and sulfite concentrations—after subtracting the corresponding abiotic controls—in cultures of strain S69ᵀ grown under thiosulfate disproportionation (S₂O₃D) conditions (C) and in hydrogenotrophic sulfite-containing medium (hSO₃) (D).

**FIGURE S2 |** Results of peptide *m/z* intensity normalization using a median-based method: (A) intensity-based violin plot, (B) principal component analysis (PCA) and (C) intensity distribution plot and of the different biological replicates. References: S2O3D corresponds to S_2_O_3_D; SO3R corresponds to hSO_3_; _pilexp_ refers to replicate 4.

**FIGURE S3 |** Overlapping protein groups identified across three biological replicates (R1–R3) for thiosulfate disproportionation -S_2_O_3_D- (A, B) and four biological replicates (R1–R4) for cultures carried out in hydrogenotrophic sulfite-containing media -hSO_3_- (C, D). The left diagram shows the membrane fraction (Memb), while the right diagram represents the soluble fraction (Sol).

**FIGURE S4 |** Genomic organization of the key genes discussed in this study**.**

**FIGURE S5 |** Venn diagrams illustrating proteins identified in strain S69ᵀ in soluble -Sol- and membrane -Memb- fractions under S_2_O_3_D (A) and in the hSO_3_ condition (B).

**FIGURE S6 |** Heatmap showing the relative abundance of proteins (fold change ≥ 1.5; adjusted *p*-value ≤ 0.05). A detailed list of these proteins is provided in Table S8.
